# Design, delivery, and evaluation of a knowledge translation intervention for multi-stakeholders

**DOI:** 10.1186/s43058-023-00465-9

**Published:** 2023-07-24

**Authors:** Gurprit Kaur Randhawa, Juma Orach, Agnes Black, Vivienne Chan, Naomi Potter, Jacqui Brinkman, Hélène Côté, Larry Worfolk, Darryl Knight, Ivan Leversage, Scott J. Tebbutt

**Affiliations:** 1 University of British Columbia, Vancouver, BC Canada; 2 University of Victoria, Victoria, BC Canada; 3 Providence Health Care, Vancouver, BC Canada

**Keywords:** Knowledge translation, Knowledge mobilization, Training, Patient oriented, Multi-faceted Intervention, Mixed methods approach

## Abstract

**Background:**

Knowledge translation (KT) is a key competency for trainees (graduate students and post-doctoral fellows), the new generation of researchers who must learn how to synthesize, disseminate, exchange, and ethically apply knowledge to improve patient and health system services, products, and outcomes. KT training is a key enabler to support KT competency development. Yet, there is a dearth of research on the design, delivery, and evaluation of KT training for trainees.

**Methods:**

The study applied a QUAN(qual) mixed methods approach with an embedded experimental model design. A heart and lung patient was also recruited to participate as a partner and researcher in the study. A multi-faceted KT intervention for trainees was designed, delivered, and evaluated. Data were collected using surveys and focus groups. Quantitative data were analyzed using descriptive and inferential statistics in R Studio and MS Excel. Qualitative data were analyzed in NVivo using thematic analysis.

**Results:**

Participation in each KT intervention varied, with 8–42 participants attending KT webinars, 61 attendees in the Three Minute Thesis (3MT) Competition Heat, and 31 participants in the Patient & Public Forum. In total, 27 trainees and 4 faculty participated in at least one of the KT webinars. Trainee participants reported satisfaction, as well as statistically significant increases in 10/13 KT competencies after receiving one or more components of the KT intervention. Additionally, participating faculty, patients, and the public were satisfied with the intervention components they participated in. Several challenges and facilitators were also identified to improve the KT intervention.

**Conclusions:**

The KT intervention is a promising initiative that can be adopted and adapted across various post-secondary settings to support trainees’ competency development in KT. This evaluation demonstrates that trainees will respond to opportunities for KT training and that capacity for KT competencies can be advanced through a multi-faceted intervention that involves trainees, faculty, patients, and health system collaborators in its design and delivery. This evaluation study contributes the design and results of a novel KT intervention for multi-stakeholders.

**Trial registration:**

N/A.

**Supplementary Information:**

The online version contains supplementary material available at 10.1186/s43058-023-00465-9.

Contributions to the literature • Bridges the gaps in KT training for multi-stakeholders, especially trainees, through an innovative, multi-faceted KT intervention that includes diverse KT stakeholders in its design and delivery. The KT intervention and findings from this novel patient-oriented research study can be adopted and adapted across various post-secondary settings to advance trainees’ KT competency development.

• Demonstrates that trainees will improve KT competencies through KT training that is tailored to their learning needs, varied in delivery format, and includes diverse stakeholders, especially patient partners.

• Advances patient-oriented research and mutual benefits for KT by including patients as both partners in intervention design and as knowledge users of the KT intervention.

## Background

Across the globe, there is increasing pressure from funding bodies and post-secondary institutions for current and emerging researchers to participate in knowledge translation [15, 28, 36]. Knowledge translation (KT) is broadly defined as a process of synthesis, dissemination, exchange, and ethically sound application of knowledge to improve the health of patients, provide more effective health services and products, and strengthen the health care system [12]. KT is also referred to as knowledge mobilization (KM), and the two terms are often used interchangeably in the literature and practice.

To support the complex, dynamic, and iterative process of KT [12], KT training has been identified as a critical success factor [16, 28, 31]. However, the time and cost of KT training are the biggest barriers to KT training [16]. In addition to many KT training initiatives that have focused on training faculty [33, 36] and health care professionals [19, 23], there is a need to incorporate KT in graduate and postgraduate training for the next generation of researchers [37]. Mishra et al. [27] identified that current graduate programs do not have organized training for critical KT skills that allow trainees to gain professional expertise and better appreciate the true added-value of their research training endeavors [27]. While trainees are increasingly identifying KT as their research discipline [7], there are few graduate programs specifically targeting KT skills [29]. A 2010 global scoping review of existing KT training opportunities revealed that a large proportion of the identified KT training targeted “trainees of KT research,” and specifically graduate students or post-doctoral fellows [2]. These KT training offerings were often delivered as full courses for trainees instead of short courses, modules, seminars, or workshops [2]. Given trainees often have very large academic and research workloads, the full course format limits the accessibility of KT training. At the same time, there is little research on how to build KT skills in trainees [1] and the effective design of KT courses is still nascent [17]. A recent study by Gaid et al. [11] has identified 51 educational training offerings for rehabilitation professionals or graduate students enlisted in a rehabilitation program in Canada. The study found that only 55% of the training offerings focused on KT skills while 53% provided foundational knowledge on KT, revealing a lack of comprehensive educational training to prepare participants with KT competencies [11].

While there is evidence of many KT training offerings for trainees in North America, there is a dearth of research on the design, delivery, or evaluation of KT training for graduate and post-doctoral trainees [31, 35], further hindering the development of effective and organized KT training curriculum for trainees. A recent scoping review found that little is known about how health research trainees engage in integrated KT (iKT), whereby researchers make research more useful through research partnerships [6].

The University of British Columbia (UBC) Centre for Heart Lung Innovation (HLI) is a translational heart, lung, and critical care research center located at St. Paul’s Hospital in Vancouver, BC, Canada. The vision of HLI is to prepare and empower trainees to succeed after graduate school, becoming the next generation of scientific experts, leaders, professionals, communicators, collaborators, and advocates who make a positive difference in the world. Given that many of HLI’s existing training programs focus on publishing or presenting research findings to scientific peers, HLI’s trainees identified a need for KT training in 2019 following participation in a “Career Paths for Researchers” (CPR) program offered at UBC. To advance KT at HLI and also address gaps in KT training research, it was critical for HLI to design, deliver, and evaluate a KT intervention for trainees that is delivered through learning modules, seminars, workshops, coaching sessions, and applied practice opportunities instead of a full course format.

The purpose of this research study was to: (1) design, deliver, and evaluate a KT training curriculum for HLI trainees who engage in basic, translational, and clinical research, and (2) connect HLI trainees with external organizations, patient and family partners (PFP), and resources within UBC, Providence Health Care (PHC), and other local academic health institutes to advance iKT.

### Research questions

The primary and secondary research questions for this project are outlined below in Table 1.

**Table 1:** Study research questions

Primary research question	(1) What are the effects of the KT training curriculum on trainees in terms of:
	(a) Satisfaction with the KT training curriculum?
	(b) Confidence in carrying out KT activities?
	(c) Knowledge, skills, and attitudes in carrying out KT in their research projects?
	(d) Identifying ways to build KT into their research projects?
	(e) Communication, collaboration, and networking skills in working with research stakeholders?
	(f) Level of engagement in their graduate and postgraduate studies?
	(g) Competitiveness for the current job market?
	(h) Likelihood of applying to UBC Public Scholars Initiative (PSI) and other “Re-imagining the PhD” opportunities?
Secondary research questions	(2) What are the effects of the KT training curriculum on faculty in terms of:
	(a) Satisfaction with the KT training curriculum?
	(b) Engagement with stakeholders to enhance knowledge mobilization for their own research?
	(c) Perceived support in providing a well-rounded training environment for their students?
	(3) How satisfied are partner organizations in their collaboration with the project team?
	(4) How satisfied are patients with the Patient & Public Forum?
	(5) How satisfied are the public with the Patient & Public Forum?
	(6) How engaged are patients in the Patient & Public Forum?
	(7) How engaged are the public in the Patient & Public Forum?

## Methods

### Study design

The study applied a QUAN(qual) mixed methods approach with an embedded experimental model design. In this design, priority is established by the quantitative, experimental methodology with qualitative data collected to supplement the methodology [8]. A two-phase design was used to collect qualitative data before, during, and after the intervention to explain the results of the intervention and to follow up on the experiences of study participants [8]. A heart and lung patient was also recruited to participate as a partner and researcher in the study, including the design, delivery, and evaluation of the KT intervention. As such, this is also a patient-oriented research (POR) study.

### Study population and recruitment

Three categories of participants were recruited for the study, as described below. Study recruitment was conducted from November 2021 to June 2022.

- *Category A*: For the primary research question, trainees from the UBC Centre for Heart Lung Innovation (HLI) were recruited to participate in the KT program for this study. This included Master’s students, MD/PhD students, PhD students, and Post-doctoral fellows. For the secondary research question, HLI faculty were recruited to engage in the KT program for this study. Prospective participants in Category A were identified through the UBC HLI, which had a list of all trainees and faculty. Category A participants were recruited through third party recruitment. The study participation opportunity was advertised through the HLI, which had an email distribution list of all trainees and faculty at HLI. Prospective participants received an email with the study invitation.
- *Category B*: For the secondary research question, patients with a heart or lung condition and members of the general public were recruited to participate in Patient & Public Forums for this study. Category B participants were identified through various networks such as the Patient Voices Network and the BC SUPPORT Unit for Patient & Public Forums. Recruitment was done using an email invitation and public posting that was advertised through HLI, the Patient Voices Network, and Providence Health Care. Public posters were posted at St. Paul’s Hospital.
- *Category C*: For the secondary research question, collaborators were recruited to provide KT training for this study. Category C participants were identified through the training curriculum design. They included speakers and facilitators recruited to deliver the training program sessions. Recruitment was conducted directly using a study invitation delivered through email.

### Study inclusion criteria

To be eligible for inclusion in the research study, individuals needed to meet the inclusion criteria for their respective participation category, as outlined below.

#### Category A:

- Be an HLI trainee (Master’s student, MD/PhD student, PhD student, or Post-doctoral fellow) or faculty.
- Be interested in implementing and applying the training in their research practice.

#### Category B:

- Be a patient with a heart or lung condition or a member of the general public.
- Be able to speak, understand, and write English.
- Be interested in learning about new health care research.
- Be willing to provide feedback on how researchers can better communicate research findings to patients and the public.
- Be willing to share their experience with participating in the Patient & Public Forum.

#### Category C:

- Be an invited speaker/facilitator for the KT training program at UBC HLI.
- Be willing to share their experience in collaborating with the KT training project team.
- Be willing to provide feedback on opportunities to improve KT collaboration.

For Category A and C participants, exclusion from the study occurred for individuals who did not have access to the internet or Zoom video conferencing software (San Jose, USA). Category B participants were excluded from the study if they were unable to join the Patient & Public Forums in-person or via Zoom.

### Intervention

The study intervention was multi-faceted and varied depending on the type of participant.

Category A participants received at least one or more of the following interventions: (1) between 1–11 KT training sessions, (2) opportunity to participate in or attend a Three Minute Thesis (3MT®) Competition to practice and apply their KT competencies, (3) opportunity to participate in or attend a Patient & Public Forum, (4) coaching sessions to prepare for the 3MT Competition, and (5) coaching sessions to prepare for the Patient & Public Forum. It should be noted that due to competing trainee workloads, Category A participants were not required to receive the full intervention (i.e., KT training, 3MT Competition, and Patient & Public Forum) to participate in the study. Category B participants received the opportunity to participate in a Patient & Public Forum. Category C participants did not receive an intervention, as they were collaborators/supporters in delivering the KT training intervention.

The KT training session series was designed from August-November 2021 based on (1) a learning needs assessment conducted with HLI trainees and faculty in October 2021, (2) a review of UBC’s Kx (Knowledge Exchange) unit learning materials, (3) a review of best practices, tools, and resources for KT, (4) consultation with various KT experts in BC, and (5) feedback and suggestions from the KT Project Steering Committee. The learning needs assessment included a survey provided to all HLI trainees and faculty, and received 23 responses. The results of the learning needs assessment can be seen in Additional file 1. A training curriculum and graphic syllabus was then designed, including KT program and session-specific learning outcomes (See Additional file 2). The training content included KT in health research and health care; KT stakeholders; patient-oriented research; Indigenous peoples, stakeholder engagement; KT partnership and collaboration; KT planning; communications in KT; KT evaluation; and networking in KT. A detailed description of the KT training sessions is included in Additional file 3. Canvas (Instructure, UT, USA) was the online learning platform used to host the KT learning modules, including webinar recordings and resources.

For the first time in HLI history, a 3MT competition heat was organized specifically for HLI trainees, which took place virtually in February 2022. To support trainees in preparing for the 3MT competition, resource guides/tip sheets were shared and the KT Project Team also hosted coaching sessions to provide trainees with an opportunity to practice their 3MT presentations and receive feedback. Judges from HLI and PHC were recruited to adjudicate the 3MT competition, and prizes were advertised for the winning presentations.

The Patient & Public Forum was designed based on the format of the 3MT competition. To ensure an appropriate design for patients and the public, the design was modified based on feedback and guidance from the KT Steering Committee, including suggestions from a PFP. To support trainees with preparing for the Patient & Public Forum, resource guides/tip sheets were provided and the KT Project Team also hosted coaching sessions to provide trainees with an opportunity to practice their presentations and receive feedback prior to the Patient & Public Forum. Prizes were advertised for the winning presentations.

### Data collection

Data were collected using multiple data collection tools from December 2021 to June 2022. Table 2 below outlines the Research Question number (RQ#), evaluation metric(s), and evaluation method(s), including data collection tools.

**Table 2:** Research questions, metrics, and evaluation methods

RQ #	Evaluation metric(s)	Evaluation method
1a	Trainee satisfaction with KT training curriculum	Post-Session Survey, Post-Intervention Survey, Focus Group
1b	Level of trainee confidence in carrying out KT activities	Pre-Intervention Survey, Post-Session Survey, Post-Intervention Survey
1c	Trainee knowledge, skills, and attitudes in carrying out KT in their research projects	Pre-Intervention Survey, Post-Session Survey, Post-Intervention Survey
1d	Trainee development of KT Plan	Pre-Intervention Survey Post-Intervention Survey
1e	Trainee’s communication, collaboration, and networking skills in working with non-academic research stakeholders	Pre-Intervention Survey, Post-Session Survey, Post-Intervention Survey, Focus Group
1f	Level of trainee engagement in their graduate and postgraduate studies	Pre-Intervention Survey, Post-Session Survey, Post-Intervention Survey, Focus Group
1 g	Trainee competitiveness for KT funding opportunities and the current job market	Pre-Intervention Survey Post-Intervention Survey
1 h	Likelihood of applying to UBC Public Scholars Initiative (PSI) and other “Re-imagining the PhD” opportunities	Pre-Intervention Survey, Post-Session Survey
2a	Academic faculty satisfaction with KT training curriculum	Post-Intervention Survey, Focus Group
2b	Academic faculty engagement with stakeholders to enhance knowledge mobilization for their own research	Pre-Intervention Survey, Post-Intervention Survey, Focus Group
2c	Academic faculty’s perceived support in providing a well-rounded training environment for their students	Post-Intervention Survey, Focus Group
3	Partner Organization satisfaction with collaboration	Collaborator Survey
4	Patient satisfaction with public and patient forums	Post-Session Survey, Focus Group
5	Public satisfaction with public and patient forums	Post-Session Survey, Focus Group
6	Patient engagement in the public and patient forums	Session Attendance, Post-Session Survey, Focus Group
7	Public engagement in the public and patient forums	Session Attendance, Post-Session Survey, Focus Group

### Data collection tools

To evaluate the research questions, the Pre-Intervention Survey, Post-Intervention Survey, Post-Session Survey, Collaborator Survey, and Focus Group questions were developed in November 2021 by the research team and assessed for face validity. Due to the paucity of relevant psychometrically tested survey tools for KT training evaluation, custom data collection tools were designed to measure the research questions. The data collection tools are included in Additional file 4 and the overall data collection plan is illustrated in Additional file 5.

All surveys were self-administered online, hosted on the Qualtrics survey platform, and took 10–15 min to complete. The Pre-Intervention Survey consisted of 16 questions covering two topics: (1) demographic characteristics (two questions) and (2) self-assessment of KT competency (13 closed-ended questions on a 10-point scale and one open-ended question). Fifteen questions were mandatory to complete the survey. The Post-Intervention Survey consisted of 19 questions covering two topics: (1) demographic characteristics (3 questions) and (2) self-assessment of KT competency (13 closed-ended questions on a 10-point scale and three open-ended questions). Eighteen questions were mandatory to complete the survey. The Post-Session Survey consisted of 21 questions covering four topics: (1) demographic characteristics (two questions), (2) training session satisfaction (seven questions on a Likert scale from “Very Dissatisfied” to “Very Satisfied,” (3) self-assessment of KT competency (7 closed-ended questions on a 10-point scale), and (4) questions related to training session takeaways (four open-ended questions and one checkbox question). Sixteen questions were mandatory to complete the survey. The Collaborator Survey consisted of 11 questions covering two topics: (1) demographic characteristics (one question) and (2) collaboration satisfaction (six closed-ended questions on a Likert scale and four open-ended questions). Nine questions were mandatory to complete the survey.

The focus groups for trainees, faculty, and patients and the public included 9–13 questions and were designed to be 45–60 min in length. The following five topics were covered in all three focus groups: (1) definition of KT (one open-ended question), (2) satisfaction with the KT intervention (three open-ended questions), (3) KT intervention improvement opportunities (one open-ended question), and (5) any other information the participant would like to share (one open-ended question). Additionally, the trainee focus group included six open-ended questions related to KT competency while the faculty focus group included one open-ended question on this topic. However, the faculty focus group also included five open-ended questions about the KT competency of trainees. The patient and public focus group included two open-ended question related to patient empowerment.

### Data analysis

#### Quantitative data

Quantitative data were analyzed using descriptive statistics in MS Excel (Version 2206, Build 16.0.15330.20260) and inferential statistics (*t* tests) in Excel and R studio (Version 4.1.2). Thematic analysis was used to analyze qualitative data (free text in survey data and focus groups) in NVivo 1.6.1 (1137). Focus group data were transcribed using Zoom transcription and verified by manual review of the Zoom recording.

Descriptive statistics from the Pre-Intervention Survey, Post-Session Survey, and Post-Intervention Survey were analyzed. The means, medians, modes, ranges, and standard deviations were calculated. Differences in pre- and post-intervention KT measures for Category A participants were tested using independent Student’s *t* tests. Following multiple comparisons adjustment using the Benjamini Hochberg method, *p* values < 0.05 were considered statistically significant.

Qualitative data from free-text comments in the Pre-Intervention Survey, Post-Session Survey, Post-Intervention Survey, and focus groups were analyzed using thematic analysis. Thematic analysis allowed themes within the data to be identified [32] through the systematic process of coding, sorting, and interpreting data [24]. The four steps of thematic data analysis were followed, including (1) immersion, (2) coding, (3) categorizing, and (4) generation of themes [13]. Immersion included the repeated reading and re-reading of the qualitative data [13]. Following immersion, coding was central to the analysis process [24]. Using NVivo 1.6.1 (1137), the qualitative data were reviewed and coded. Pieces of text were coded (referred to as “chunks”) using open coding. The coding scheme was refined and extended while reviewing the transcripts. Chunks that were coded in the same way were then collated [24]. To ensure trustworthiness, all coding was reviewed by a second researcher to check agreement. To support verification of the codes, the second researcher was provided the study code book and encouraged to add additional codes, as needed. After coding, the next step in thematic analysis was categorization, which involved linking the codes to create coherent categories [13]. The final step in analyzing the qualitative data involved the identification of themes.

Quantitative and qualitative data analyses were initially conducted separately. However, findings were interpreted by corroborating quantitative and qualitative findings. As a part of QUAN(qual) mixed methods design, quantitative data findings were identified as the main data source to determine the efficacy of the intervention for Category A participants. Qualitative findings were used to supplement and explain the quantitative findings (i.e., explain the efficacy of the intervention).

## Results

### Participation

#### Participation in training sessions

Live participation in the KT Training Webinars and KT Planning Workshop varied from 8 to 42 participants for the 11 sessions, with a mean of 30.1 ± 9.9 participants per session, including a mean of 11.0 ± 3.6 trainees and 1.0 ± 0.8 faculty per session. Attendees other than Category A participants (i.e., HLI trainees and faculty) were invited to attend the KT Training Webinars, such as stakeholders from Vancouver Coastal Health and Provincial Health Services Authority.

In total, 27 trainees and four faculty participated in at least one of the live KT training sessions. Only one trainee attended all live KT training sessions. In total, 19–23 people received a certificate for completing all 11 KT training sessions in Canvas. This includes seven HLI trainees, one HLI faculty, two other HLI members, and 13 non-HLI members. Eight participants attended all training sessions live or asynchronously and attended the Patient & Public Forum. Additionally, 26 people attended all training sessions live or asynchronously, including 8–9 HLI trainees, one HLI faculty member, 1–2 other HLI staff, and 12–14 non-HLI members.

#### Participation in 3MT competition

Ten HLI trainees volunteered to participate as presenters in HLI’s 3MT Competition Heat. Of these, 3 participated in the 3MT coaching sessions. In total, 61 participants attended the 3MT thesis (54 participants via Zoom and seven in-person).

#### Participation in patient & public forum

Five HLI trainees volunteered to participate as presenters in the Patient & Public Forum. Of these, two participated in the Patient & Public Forum coaching sessions. In total, 31 participants attended the Patient & Public Forum, including 10 trainees, two faculty members, two HLI staff, five self-identified patient/public attendees, and five other unspecified attendees.

#### Participation in focus groups

Three participant role-based focus groups were conducted, in which one trainee, one patient, and two faculty participated. The length of focus groups varied from 45 to 60 min.

### Satisfaction

#### Satisfaction with training sessions

**Fig. 1:**
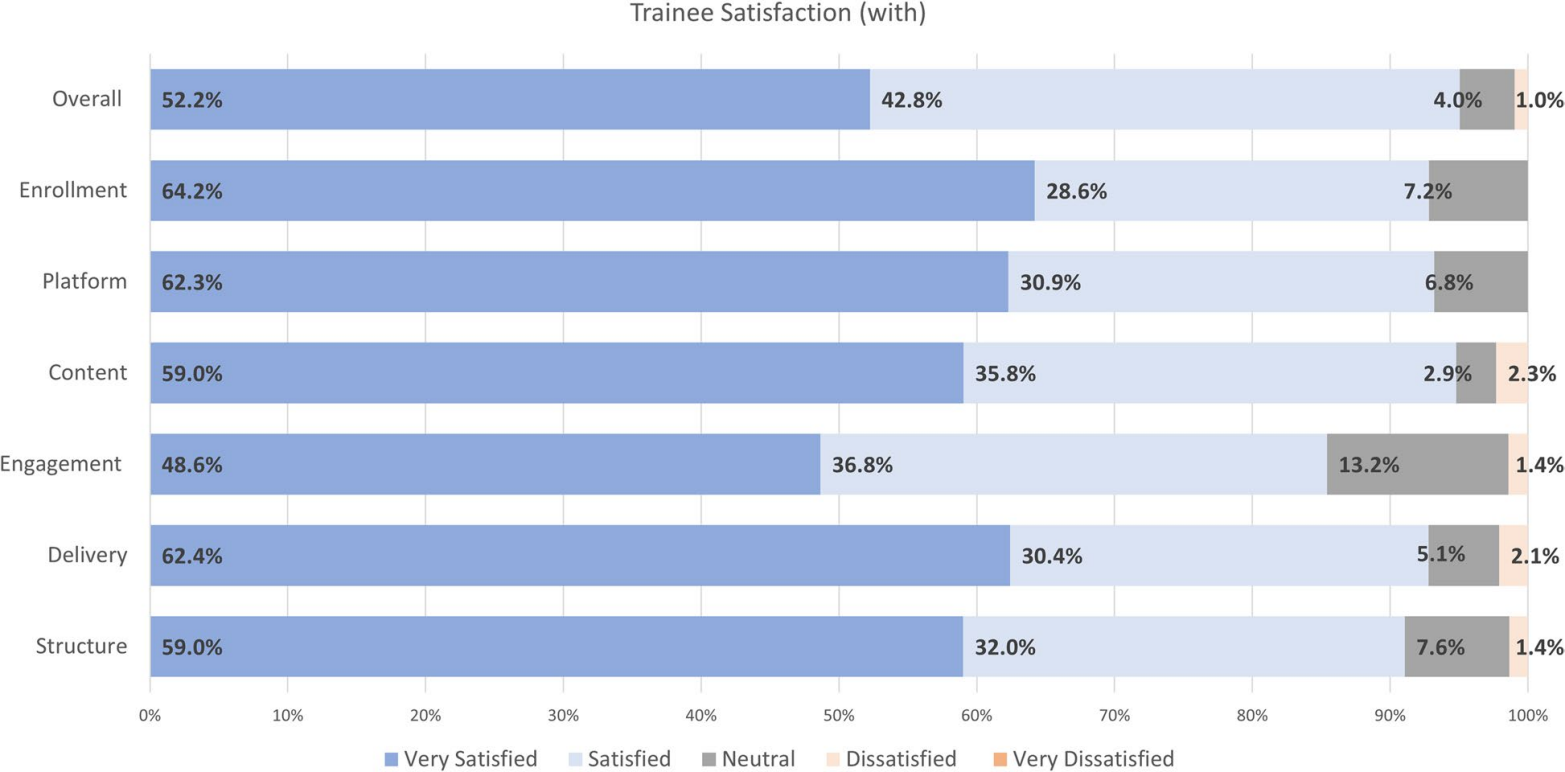
Average trainee satisfaction with training sessions (*N* = 18.4 ± 3.4)

**Fig. 2:**
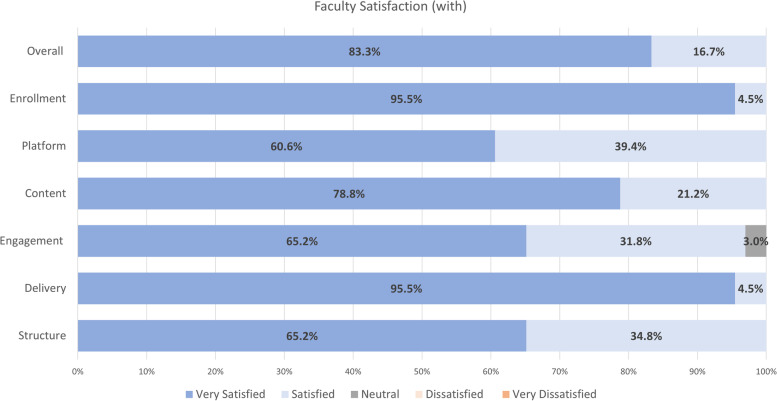
Average faculty satisfaction with training sessions (*N* = 1.9 ± 0.5)

All session participants were invited to complete a Post-Session Survey. Surveys had an average 28.1 ± 7.8 respondents, comprising 66.9 ± 7.2% trainees, 7.0 ± 1.8% faculty members, and 26.1 ± 7.8% others. The average survey response rate was 87.0 ± 13.4%, ranging from 66.7 to 100%. For trainee participants, results for session satisfaction are outlined in Fig. 1. In general, the majority (94%) of participants were very satisfied or satisfied with the KT training sessions, including the structure, delivery, engagement, content, platform, enrolment, and overall satisfaction with the KT training sessions. For faculty participants, results for session satisfaction are outlined in Fig. 2. Faculty attendees were satisfied or very satisfied with the training sessions overall, and more specifically, the structure, delivery, engagement, content, platform, and enrollment. In the focus groups, trainee and faculty participants emphasized their high satisfaction with the KT training sessions. In particular, trainee and faculty participants appreciated the training sessions that featured a panel of subject matter experts on the KT training topic.

In general, there were numerous shared themes across participant roles. In terms of the training sessions, all participant roles (trainees, faculty, and others) liked the:

- Learning design and delivery, especially the panel format of sessions;
- Subject matter;
- Hearing the patient-partner perspective and/or diverse perspectives; and
- Learning about KT resources, tools, and templates.

Trainees and faculty also highlighted the personal impact of the learning. In a focus group, one trainee participant indicated that “the panel with the patient was like super helpful” (Trainee Participant 1), and a faculty participant reported “I really benefited from this initiative, just through the planning phases and so now I have like a template to go off of and sort of utilize in my grant writing.” (Faculty Participant 1).

#### Satisfaction with patient & public forum

**Fig. 3:**
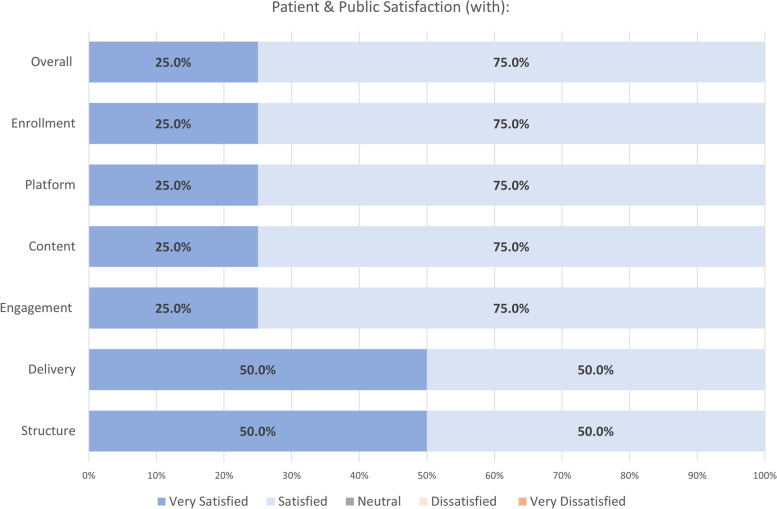
Patient and public satisfaction with Patient & Public Forum (*N* = 4)

All session participants were invited to complete a Patient & Public Forum Survey. The survey response rate was 47.83% (*N* = 11). Of these respondents, four respondents identified themselves as patient/public. Other respondents identified themselves as trainees, principal investigators, or research staff. The survey results for patient and public satisfaction can be seen in Fig. 3 below. In general, patients and public respondents were very satisfied or satisfied with the enrollment, platform, content, engagement, delivery, and structure of the Patient & Public Forum. They were also very satisfied or satisfied with the Patient & Public Forum overall.

In a focus group, a patient participant shared their experience with the Patient & Public Forum: “So I’ve been…Wow, I've been waiting for this day for years, right? To, to talk to people who are actually doing heart research about prevention of heart disease. And it's like, whoa this is about time, this is excellent, like I was, I was very happy and I felt heard. So, like when you are feeling that you are being heard. That's pretty positive, you know, development, when most of the time, you know in our society, we're not heard” (Patient Participant 1). Similarly, a trainee who presented her research at the Patient & Public Forum stated that, “…The Patient Public Forum was my favorite thing ever. Because it's like okay, this is what I want to do. This is what's important… I think just the idea that like people outside of science are going to think about things differently than me. And, like, in the patient public forum, the questions coming from the patients were like, not what I expected. The things that they cared about were not what I expected. And I think it just means like ‘Okay, you got to have those conversations’ and I really appreciated the experience with being able to do that” (Trainee Participant 1).

#### Satisfaction of partners & external organizations

In total, 19 collaborators partnered in delivering the KT training sessions. The Collaborator Survey response rate was 36.8% (*N* = 7). All respondents (100%) were very satisfied or satisfied with the support they received in preparation for their KT training session, the information received in preparation for their training session, and their overall satisfaction with the session they partnered on. All respondents were also very likely or likely to (1) recommend the partnership to someone else, (2) collaborate with the KT Project Team in the future, and (3) recommend the KT training session to someone else.

### KT outcomes and competencies

#### KT training session outcomes

**Fig. 4:**
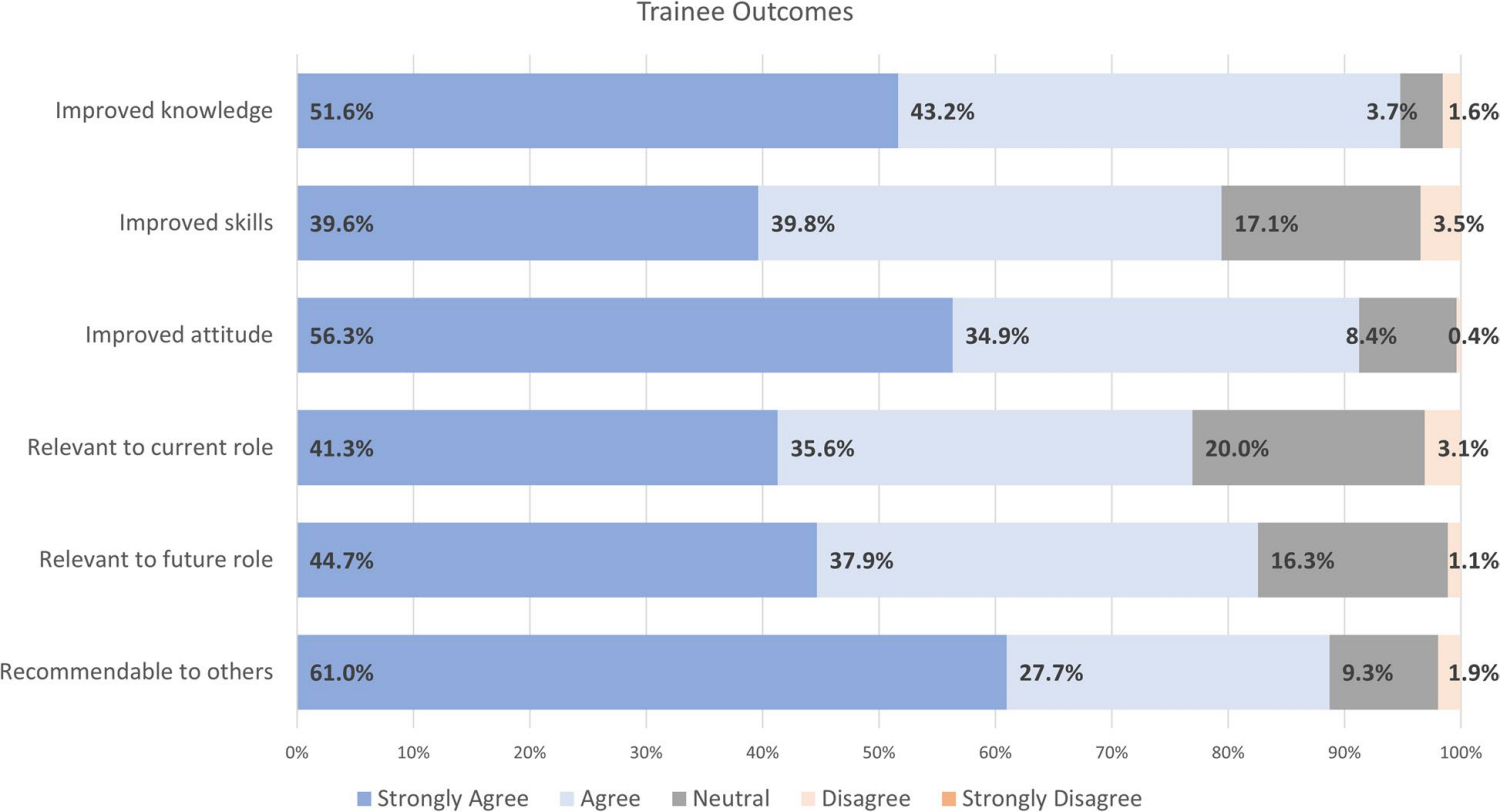
Average knowledge outcomes for trainees (*N* = 18.4 ± 3.4)

**Fig. 5:**
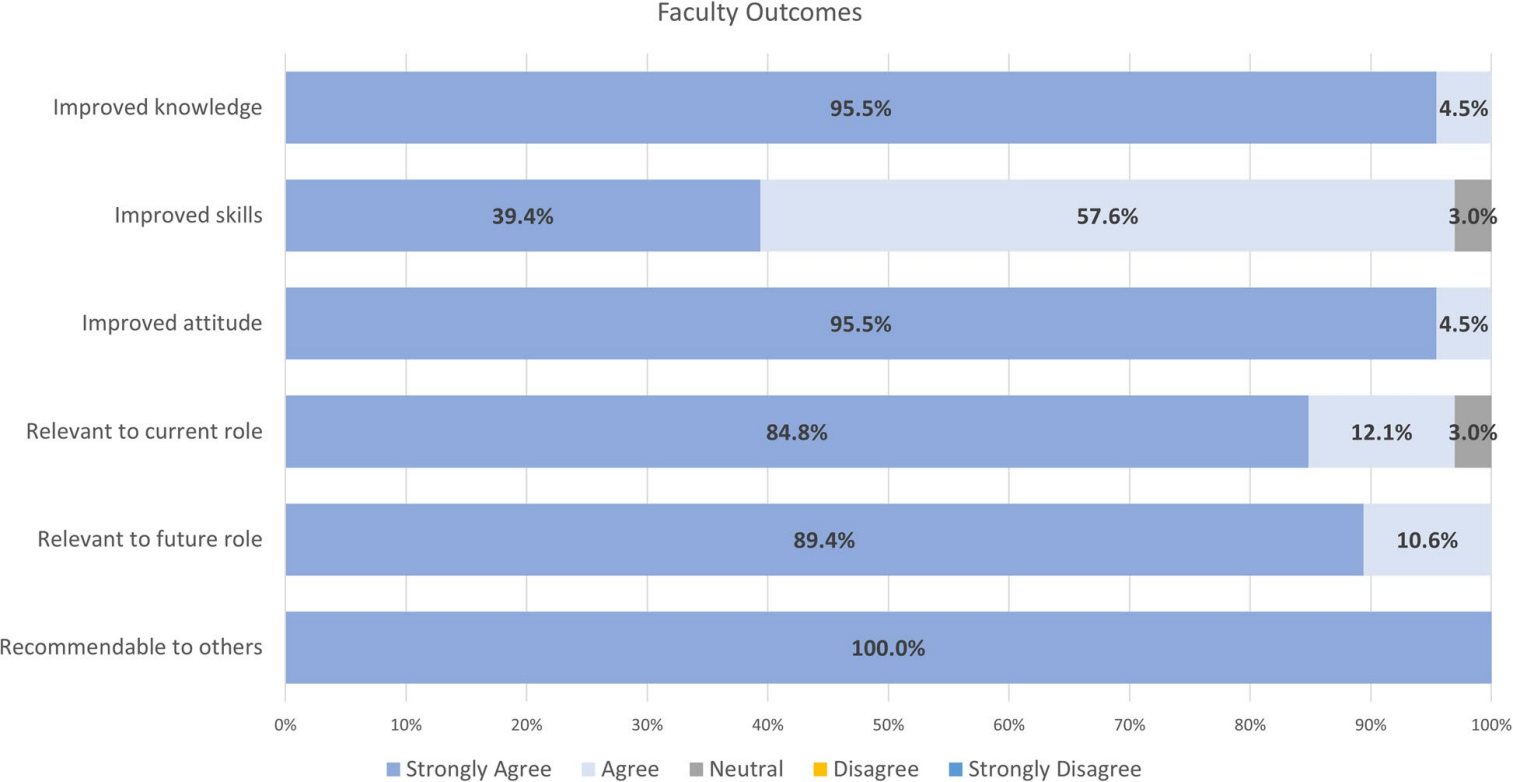
Average knowledge outcomes for faculty (*N* = 1.9 ± 0.5)

Figures 4 and 5 below depict the outcomes of the training sessions for trainees and faculty, respectively. Overall, both trainees and faculty agreed or strongly agreed that they would recommend the training to others, found the training relevant to their current and future roles, and acknowledged that the training improved their attitude, skills, and knowledge in KT.

Trainee and faculty participants reported increased KT knowledge, access to KT resources, and personal and professional inspiration to advance in their KT competencies. Faculty participants indicated that KT training ensures well-rounded training for HLI trainees. One faculty focus group participant reported, “I'm coming from a basic science research background. So it was very hard for me to even think about KT [before the KT training] because I always thought about it as reaching patients” (Faculty Participant 2). Participants also reported challenges in applying KT to their research, such as lack of a KT culture and funding. In a focus group, a trainee participant shared one of her challenges: “…my supervisor’s research program not necessarily having space for that KT conversation. And so, as a student, it's like I'm not the one who's going to be like ‘Hey, can we like talk to patients before we decide what we're going to study?’ kind of thing. So I guess that is a bit more of a like, I don’t know, cultural systemic like higher up thing that the KT program itself doesn't, you know….” (Trainee Participant 1).

#### Patient & public forum outcomes

**Fig. 6:**
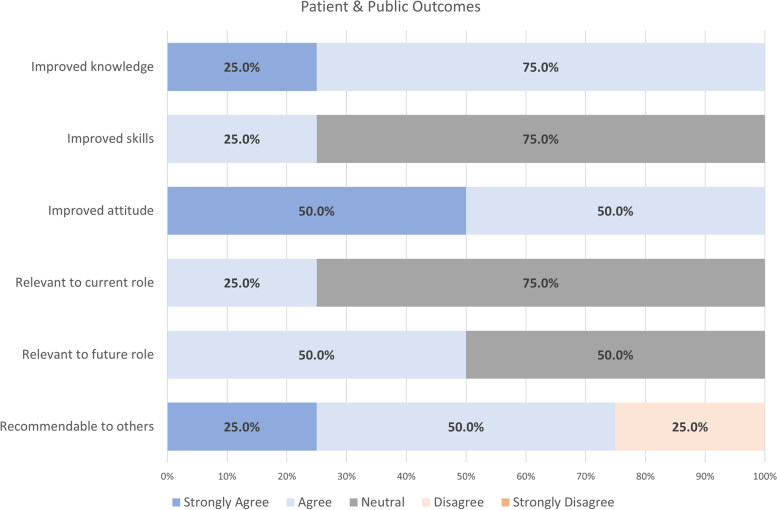
Patient and public outcomes for the Patient & Public Forum (*N* = 4)

All patient and public respondents strongly agreed or agreed that the Patient & Public Forum improved their knowledge and attitude related to KT (Fig. 6). Only 25% of respondents indicated that the forum improved their skills and was relevant to their current role. However, half (50%, *N* = 2) reported that the forum was relevant to their future role. Additionally, 75% (*N* = 3) of patient and public respondents would recommend the forum to others. In a focus group, a patient participant reported “I definitely felt heard and stuff and learned something. Yeah, [the Patient & Public Forum] was very valuable in that regard” (Patient Participant 1).

#### Overall KT intervention outcomes

**Fig. 7:**
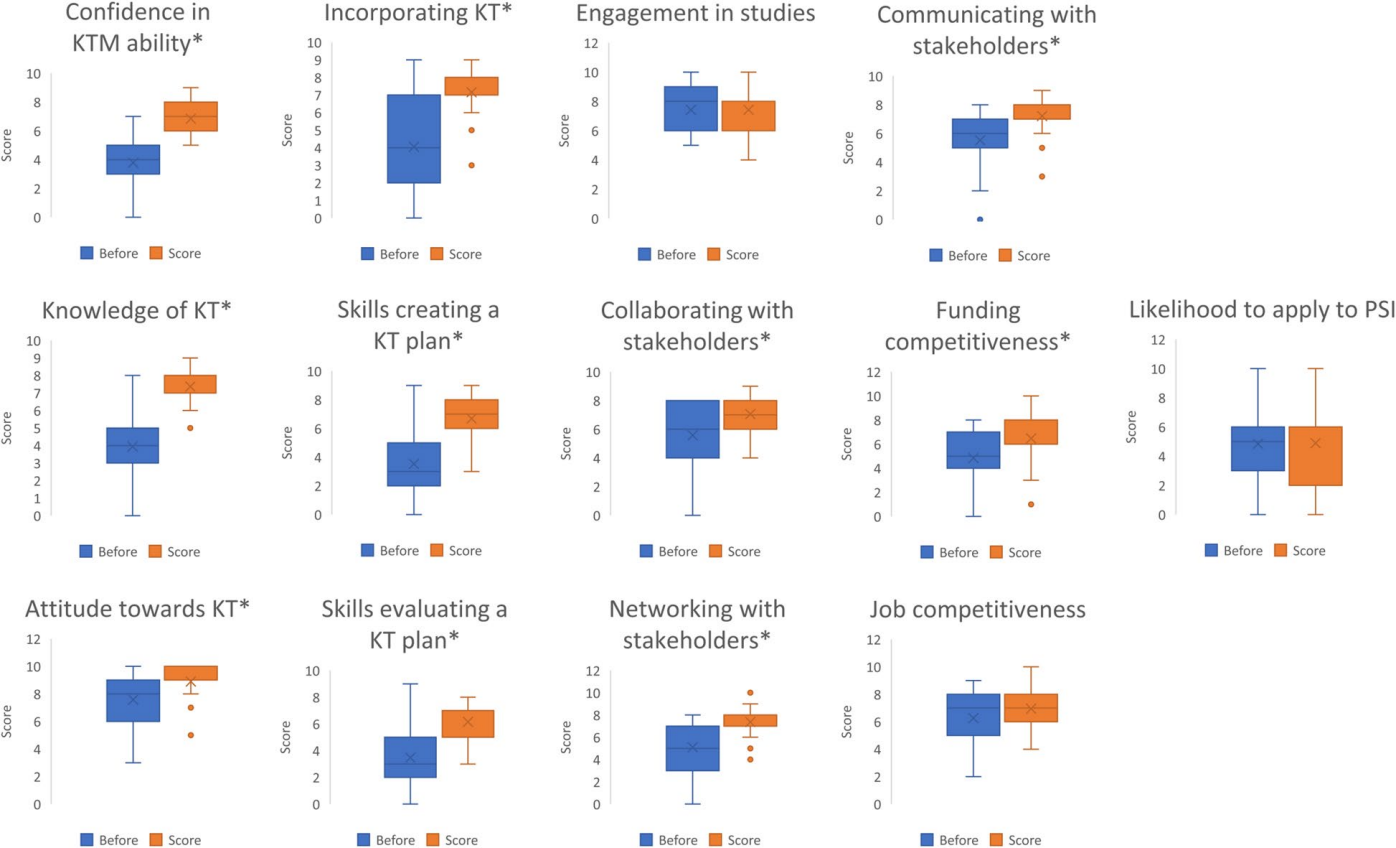
Comparisons of scores before and after the training program. Groups were compared using Student t tests and adjusted for multiple comparisons using the Benjamini Hochberg 5% false discovery rate; asterisks (*) indicate *P* < 0.05

In total, 27 trainees received the partial intervention (e.g., participation in one or more of the KT training sessions, 3MT Thesis, or Patient & Public Forum). Nineteen (19) trainees completed the Pre- and Post-Intervention Surveys, 11 of whom completed both surveys. Figure 7 illustrates the pre- and post-intervention scores for KT competencies for trainees who attended one or more of the KT training sessions.

The results reveal statistically significant increases in scores in all KT competency categories except “engagement in graduate studies,” “job competitiveness,” and “likelihood to apply for Public Scholars Initiative (PSI) opportunities”. Notably, “attitude” data in the post-intervention data were not normally distributed. These results indicate that the KT Training Intervention had a positive effect on trainees’ perceived: (a) Knowledge of KT; (b) Attitude towards KT; (c) Confidence in KT ability; (d) Skills in creating a KT plan; (e) Skills in evaluating a KT plan; (f) Communicating with stakeholders; (g) Collaborating with stakeholders; (h) Networking with stakeholders; (i) Incorporating KT into their work; and (j) Grant funding competitiveness.

In a focus group, a trainee faculty shared that the overall KT intervention made her more excited about her research, and she also shared her key takeaway: “…what I took away from the KT program was more like okay, it's important to have these [KT] conversations and the stakeholders that you talk to are often going to have a different perspective than you so make sure you’re ready to hear it” (Trainee Participant 1).

## Discussion

The evaluation of the KT training program demonstrates that a multi-faceted KT intervention for trainees is effective in improving most KT competencies in a Canadian post-secondary institution, while fostering partnership between vital KT stakeholders. Overall, the majority of trainees were satisfied or very satisfied with the KT training sessions, including the structure, delivery, engagement, content, platform, enrolment, and overall satisfaction with the KT training sessions. These findings are aligned with a recent scoping review on academic initiatives to advance KT through capacity-building interventions for graduate students [18], which found that interventions increased knowledge attainment, ability to implement evidence, productivity, and satisfaction for graduate students. Additionally, the findings reflect the importance of including guest speakers in KT training to increase trainees’ competency development, empowerment, and inspiration [5]. Further, our findings illuminate trainees’ desire for including KT training into their research and their understanding that the training will improve competitiveness for employment, as found in a KT study of Australian undergraduate and postgraduate students [30]. However, in the present study, it should be noted that there were no statistically significant differences for “engagement in graduate studies,” “likelihood to apply for PSI,” or “job competitiveness”. This may be explained by the limited hands-on application of the KT training to trainees’ own research during the training. Additionally, the opportunity to apply for PSI is restricted to PhD students only, which may have limited trainee eligibility. Hands-on application of the KT training was scheduled in the later stages of the KT intervention, which may have detrimentally affected trainees’ competencies in these three areas. To address this potential barrier, it is recommended that the KT Workshop, 3MT Competition Heat, and Patient & Public Forum be scheduled earlier in the KT intervention. As suggested by [4], leadership opportunities should also be included in the KT training to improve professional outcomes and maximize the impact of the KT training.

Overall, the majority of participating faculty were satisfied or very satisfied with the KT training, including the structure, delivery, engagement, content, platform, enrolment, and overall satisfaction with the KT training. Given the low participation of faculty in the KT intervention and faculty focus group, study results are limited in evaluating faculty’s engagement with stakeholders to enhance KT for their own research, perceived support in providing a well-rounded training environment for their students, and satisfaction with trainees’ scholarly performance. However, the faculty focus group revealed: (1) the KT Training Intervention enhanced KT for their own research, (2) The KT Training Intervention contributed to a well-rounded training environment for trainees, and (3) Faculty need more KT supports (e.g., KT Specialist to support grant writing, customized KT learning for specific research areas) to provide a well-rounded training environment and KT culture for their trainees. Diner et al. [9] also previously found that KT training needs to be tailored to address specific learner needs and that trainees need role models to demonstrate KT competencies. Faculty respondents noted the low faculty participation and highlighted time constraints as a major challenge. This is consistent with previous research identifying lack of time as a barrier to KT training [16]. Further, this finding may help to explain the challenges that trainees experienced in the present study due to limited structures, infrastructure, and time to do KT,this is similar to earlier findings on trainees’ self-reported challenges in KT [22].

Collaborators/partner organizations were satisfied or very satisfied in collaborating with UBC HLI in the KT training. Most would recommend the partnership with HLI and KT training to someone else, as well as collaborate with HLI again in the future. Notably, many training participants highlighted the significant benefits of having collaborators participate in the delivery of the KT training. This finding reinforces the mutual benefits of collaboration and partnership in KT, including the building of “productive interactions” that are prerequisite for universities to advance KT and relationships between researchers and knowledge users [10]. Further, it emphasizes the role of partnership in advancing the science of KT while developing new scholars [12], and also exposes trainees to new perspectives and modes of inquiry [20] that are necessary to advance KT [7]. Additionally, the collaboration component of the KT intervention in the present study may help address the barrier of researcher preparation for engaging in collaborative partnerships [3]. As suggested by Strauss et al. [34], collaboration partnerships may help increase the sustainability of KT training. Recent findings from the COVID-19 pandemic also reinforce the need for providing trainees with KT training on how to develop and sustain equitable collaborative research partnerships that meet and adapt to the needs of knowledge users [25].

Patient and public participation in the KT intervention is a significant contribution of this study to the literature. Trainees were deeply appreciative of the opportunity to share their research directly with the knowledge users ultimately impacted by their research: patients and the public. This underscores the importance of patients being a primary target audience for clinical research, in addition to healthcare practitioners, local administrators, policy makers, and industry [14]. Moreover, patient and public participation in our KT intervention addresses the gap of including patients and communities in KT, such as research presentations [31]. Both the KT intervention and our patient-oriented research approach to the present study advances ‘authentic engagement’ by involving patients/individuals and/or communities in all phases of the research process [31]. Additionally, the present study addresses the challenges related to researcher preparation in building credibility and acting as a messenger for KT, especially with non-academic audiences [14]. For example, both the 3MT Competition and Patient & Public Forum served as training opportunities for trainees to build credibility with patients and the public as messengers of KT.

In addition to the learning and satisfaction benefits for trainees, the participating patients and public were satisfied with the Patient & Public Forum, and strongly agreed or agreed that the Patient & Public Forum improved their knowledge and attitude related to the KT topics. They were also very satisfied or satisfied with the enrollment, platform, content, engagement, delivery, and structure of the Patient & Public Forum. In a focus group, one patient expressed how easy it was to participate in the virtual forum, and also reiterated their knowledge gained and sense of engagement in the Patient & Public Forum. Specifically, the participant “felt heard as a patient,” learned something, and felt empowered given their ability to speak during the forum. The participant mentioned the lack of opportunities like the Patient & Public Forum where patients can provide input. These findings reveal that the Patient & Public Forum increased more knowledge and attitude-based outcomes than skill and application-based outcomes and that patient and public respondents are likely to recommend the Patient & Public Forum to others. Further, our study suggests that Patient & Public Forums may increase ‘mutual learning’ for researchers and knowledge users [21]. As such, both the trainees and patients had expressed their learning discoveries from presenting at and attending the Patient & Public Forum, respectively.

### Study limitations

There were numerous limitations of this study, including training design, delivery, and participant recruitment and data collection for the 3MT Competition Heat, the Patient & Public Forum, and focus groups. Due to the COVID-19 pandemic, and especially the Omicron-variant outbreak during the training period, delivery had to be shifted to online platforms. This may have limited participant engagement and interest in participation. To meet the project timeline, training webinars were delivered over an aggressive weekly timeline. This required considerable commitment from participants and was a barrier to accessing the training, as noted by a faculty participant.

In terms of scheduling of the intervention, the 3MT Competition Heat was held before all the KT training webinars were delivered. This was done to facilitate presenters’ participation in subsequent 3MT Competitions at UBC. Scheduling the competition earlier may have increased hands-on practice of KT competencies and may have further increased trainee engagement. The timeline for patient and public recruitment for the Patient & Public Forum was also short. Additionally, the forum was delivered virtually with the option to attend in-person at St. Paul’s Hospital in downtown Vancouver only. There were significant challenges with recruiting trainee, faculty, and patient participants for the focus groups. Participation incentives were only available for patients. While scheduling was adjusted whenever possible to meet participant needs, the academic timing of the focus groups conflicted with the ability of trainees and faculty to attend the focus groups. As a result, there was a very small sample size for the focus groups as participants had limited availability to participate.

Due to funding and project time limitations, psychometric testing of the survey tools was not possible. Given that all the survey tools rely on self-assessment, the self-reported and subjective nature of the data is a limitation. The Hawthorne Effect may have contributed to the positive results in this intervention study [38]. According to the Hawthorne Effect, awareness of being studied may impact the behavior of study participants [26]. In the present study, trainees reported on their KT competencies at two time points, which suggests potential for the Hawthorne Effect. The intervention was also very large with multiple components with 11 KT Webinars and a Workshop, the 3MT Competition Heat and coaching sessions, and the Patient & Public Forum and coaching sessions. As such, there was lower participation in all the intervention components. Additionally, the analysis was limited by a relatively small sample size.

## Conclusions

The KT intervention is a promising initiative that can be adopted and adapted across various post-secondary settings to support trainees’ competency development in KT while fostering the engagement of patients and the public in research. This evaluation demonstrates that trainees will respond to opportunities for KT training and that capacity for KT competencies can be advanced through a multi-faceted intervention that involves trainees, faculty, patients, and health system collaborators in its design and delivery. It illustrates the feasibility and value of engaging with diverse KT stakeholders, such as patient-public partners and KT specialists, as collaborators and participants in the design and delivery of the intervention. This evaluation study contributes to the implementation science literature by providing the design and results of a novel KT intervention for trainees that includes KT seminars, a KT workshop, 3MT Competition Heat, Patient & Public Forum, and KT presentation coaching sessions. The study findings will help inform the design, delivery, and evaluation of future KT interventions.

## Supplementary Information

**Additional file 1:** Knowledge Translation Needs Assessment.

**Additional file 2:** KT Training Curriculum.

**Additional file 3:** KT Training Sessions Summary.

**Additional file 4:** Data Collection Tools.

**Additional file 5:** Data Collection Plan.

## Data Availability

The datasets generated and/or analyzed during the current study are not publicly available due to the limitations of the research ethics protocol but are available from the corresponding authors on reasonable request.

## Abbreviations

HLI: UBC Centre for Heart and Lung Innovation
KT: Knowledge translation
PFP: Patient and family partner
3MT: Three Minute Thesis competition
