# Supplementary material for: Design, delivery, and evaluation of a knowledge translation intervention for multi-stakeholders
Source: Implement Sci Commun. 2023 Jul 24;4:85. doi: 10.1186/s43058-023-00465-9 (PMC10364428; doi:10.1186/s43058-023-00465-9)
Supplement: Supplementary file 1 — Additional file 1: Knowledge Translation Needs Assessment. [file 43058_2023_465_MOESM1_ESM.pdf]

Knowledge Translation Needs Assessment

Questions Responses 23 Settings

23 responses

Accepting responses

Summary Question Individual

1. Please indicate your role:

23 responses

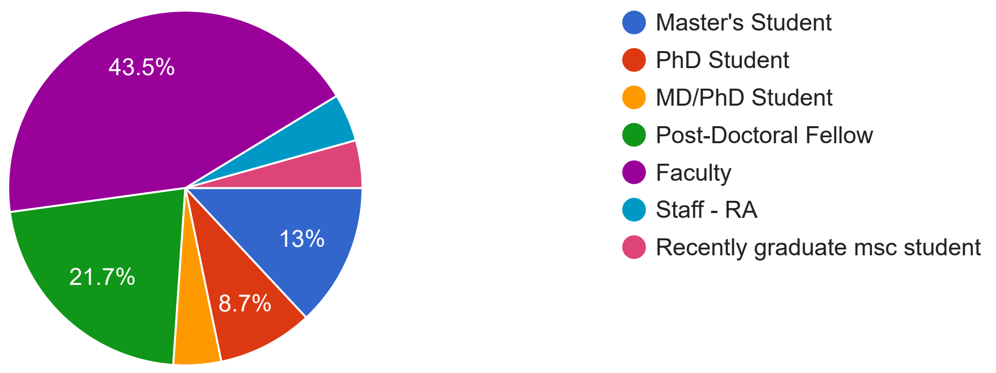

2. Please indicate which year you are in your program

23 responses

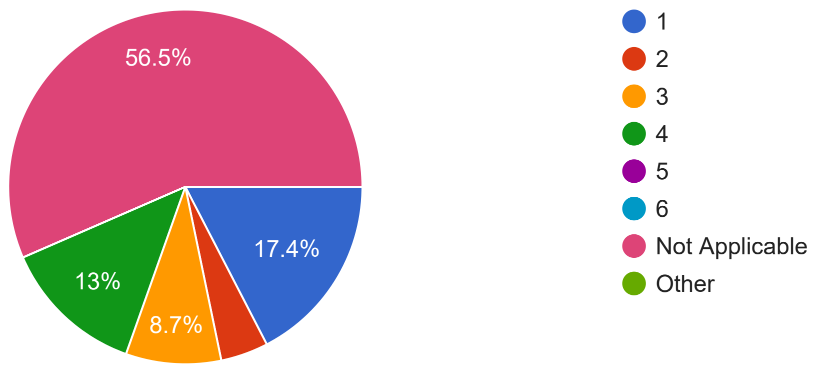

3. Please rate your confidence in carrying out knowledge translation activities.

23 responses

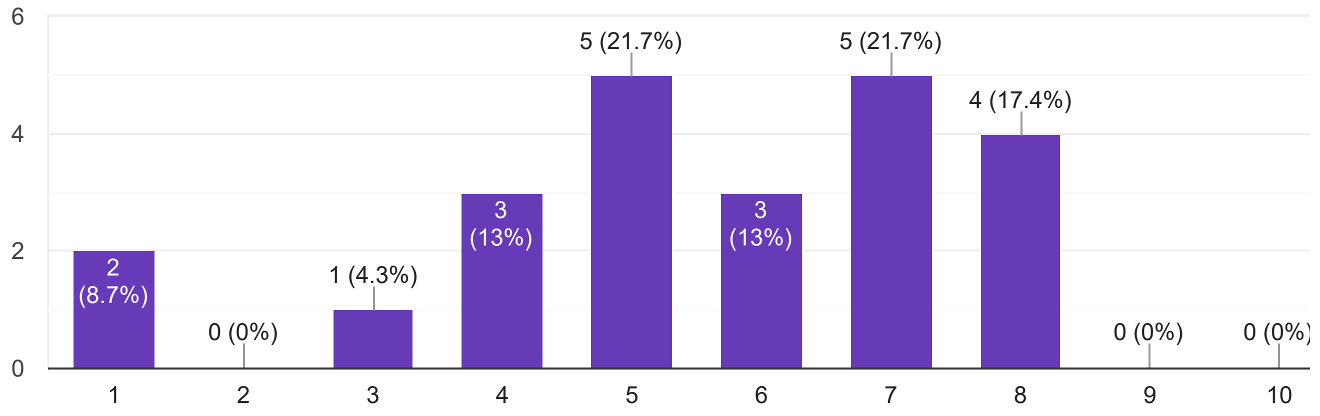

4. Please rate your knowledge on the topic of knowledge translation.

23 responses

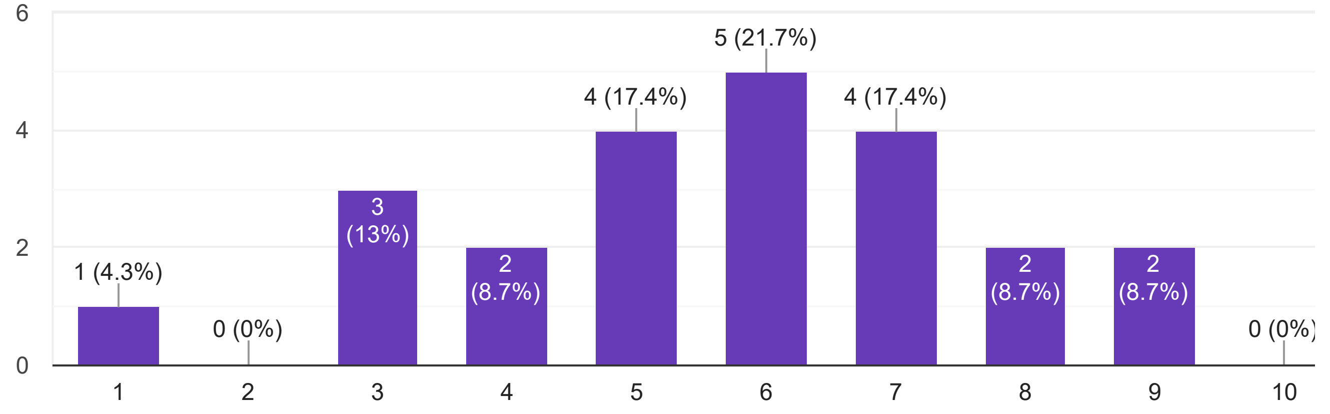

5. Please rate your knowledge of available knowledge translation resources.

23 responses

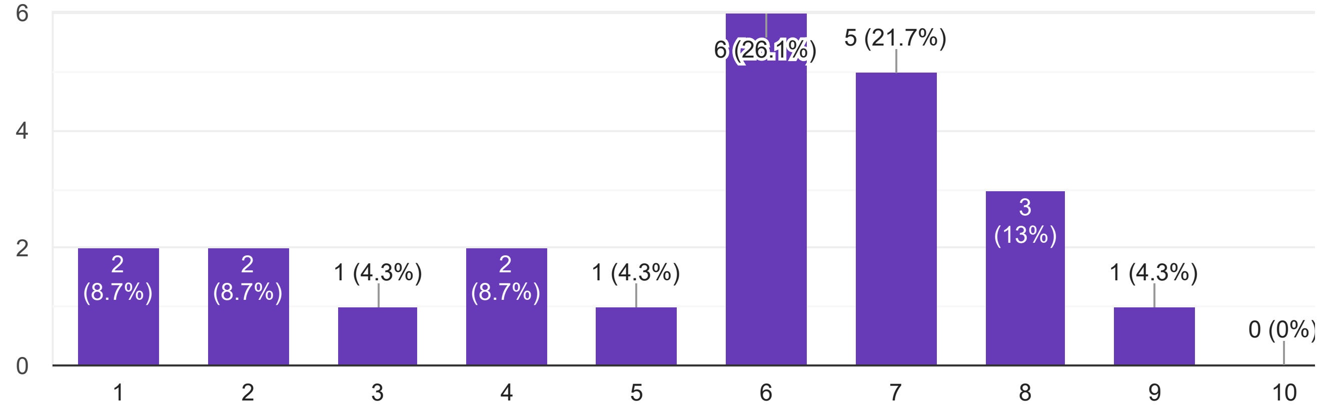

6. Please rate your skills in knowledge translation.

23 responses

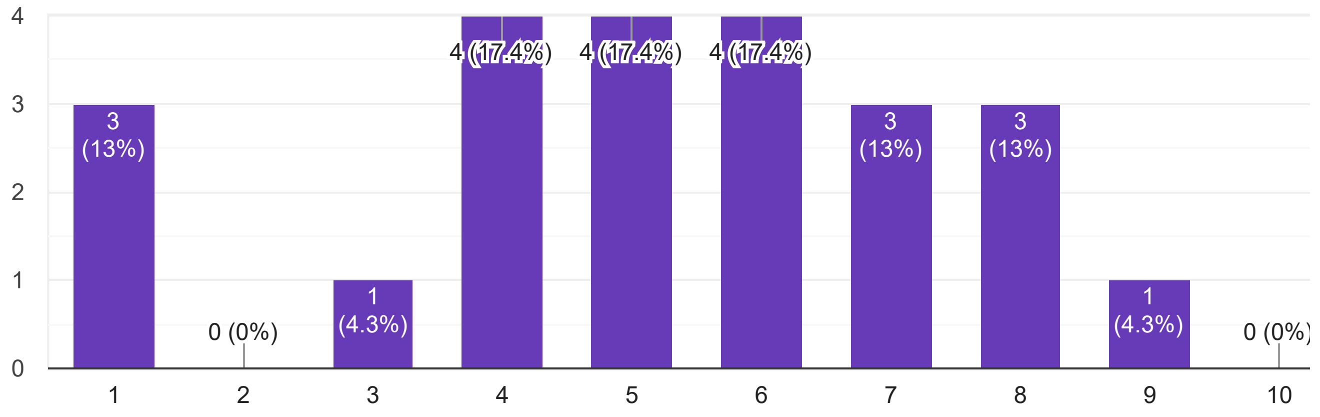

7. Please rate your experience in knowledge translation.

23 responses

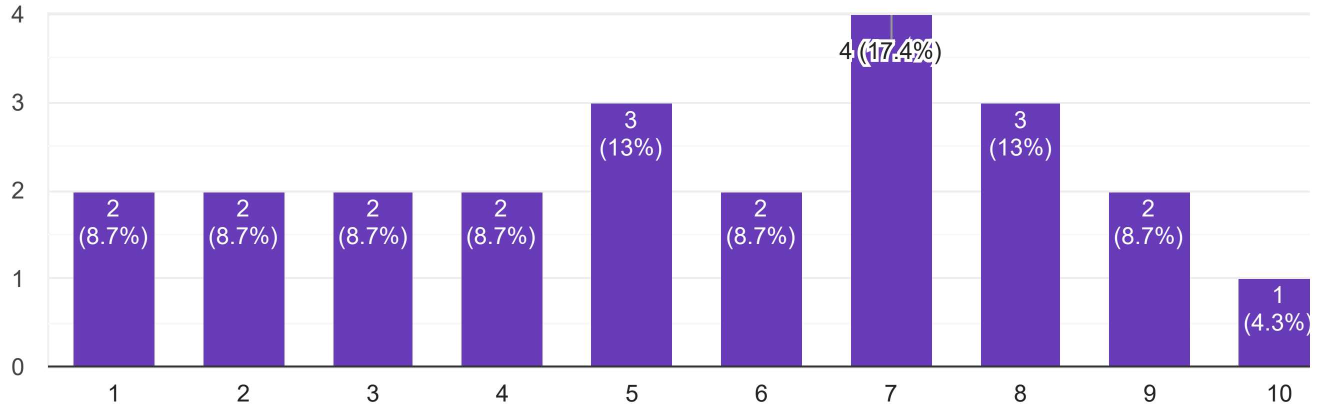

8. Please rate your ability to identify ways to build knowledge translation into your research projects.

23 responses

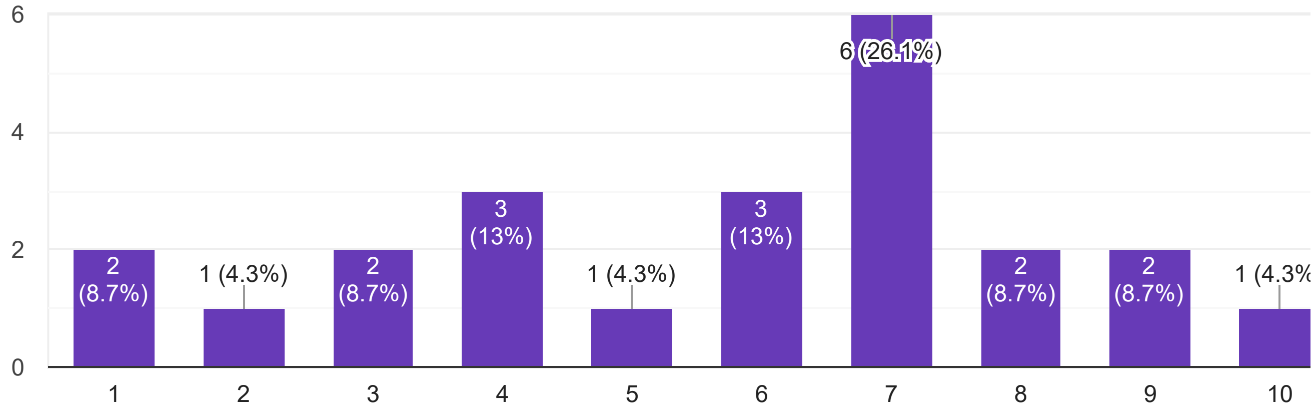

9. Please rate your communication skills in working with non-academic research stakeholders.

23 responses

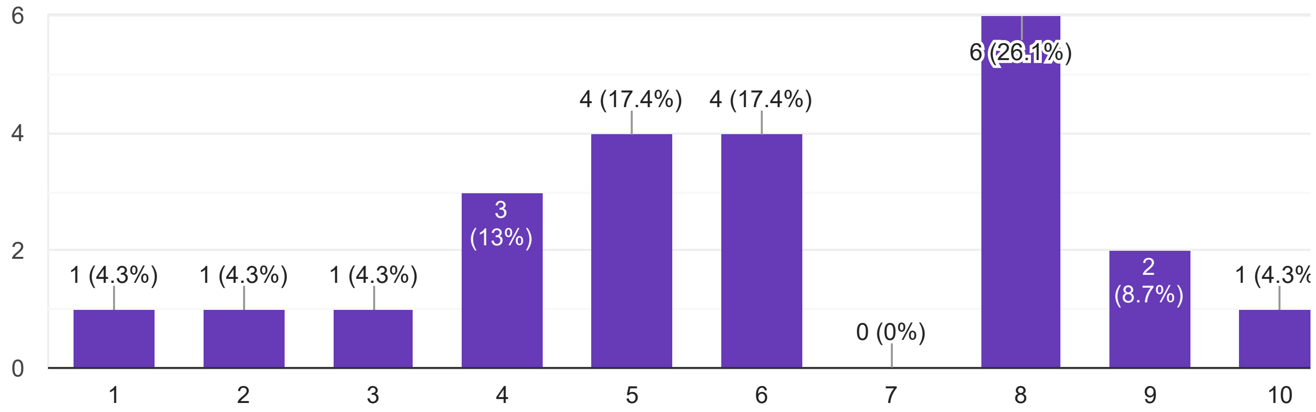

10. Please rate your collaboration skills in working with non-academic research stakeholders.

23 responses

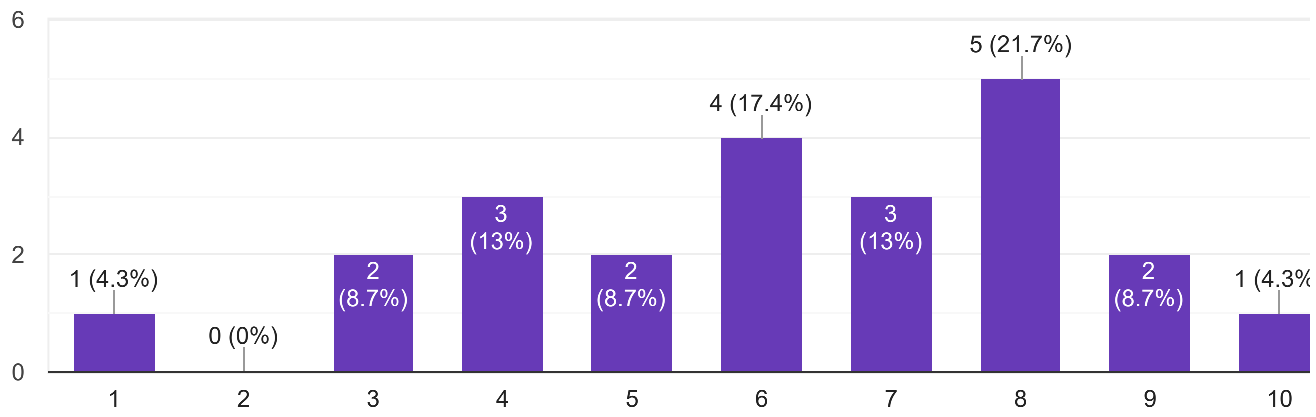

11. Please rate your networking skills in working with non-academic research stakeholders.

23 responses

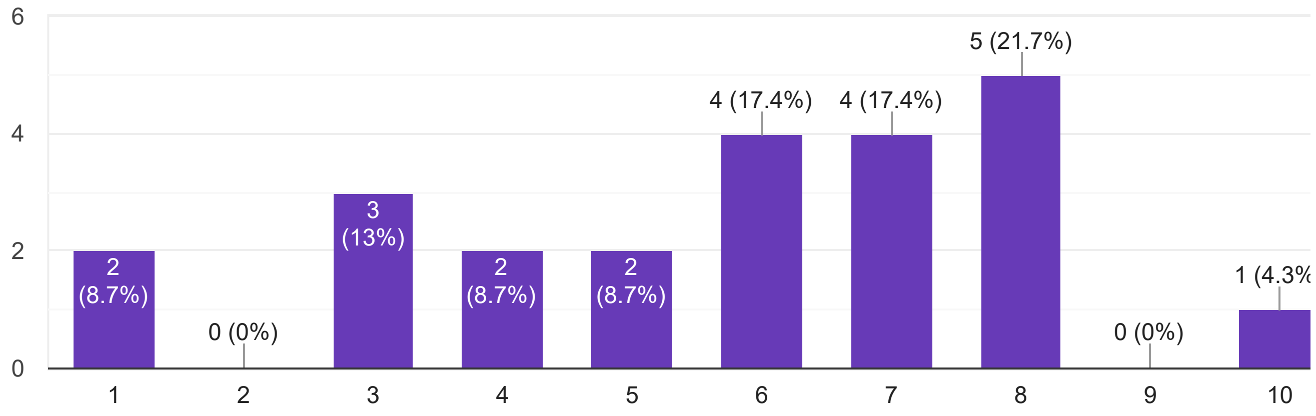

12. [TRAINEES ONLY] Please rate your level of engagement in your graduate and post-graduate studies.

11 responses

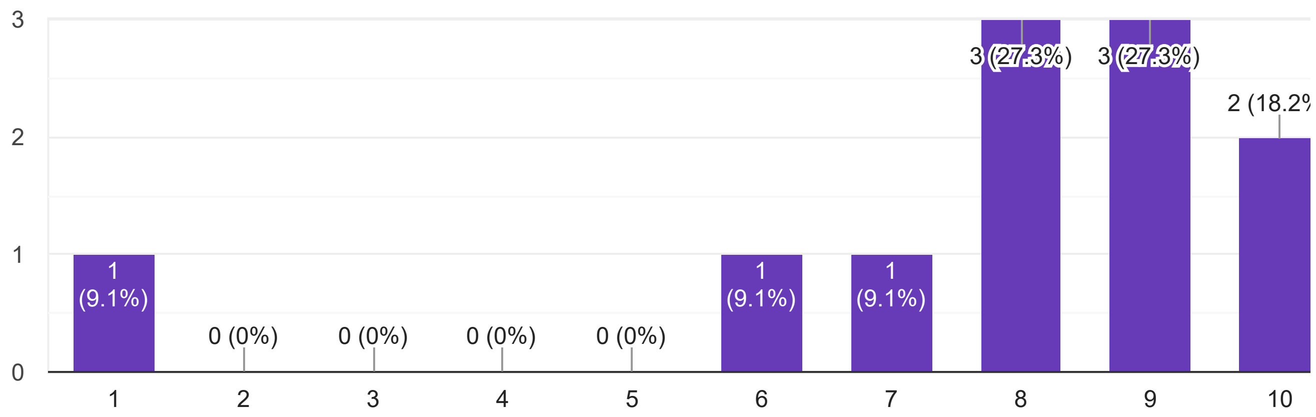

13. Please rank in order of importance knowledge translation activities for trainee research (1 = Most Important, 6 = Least Important):

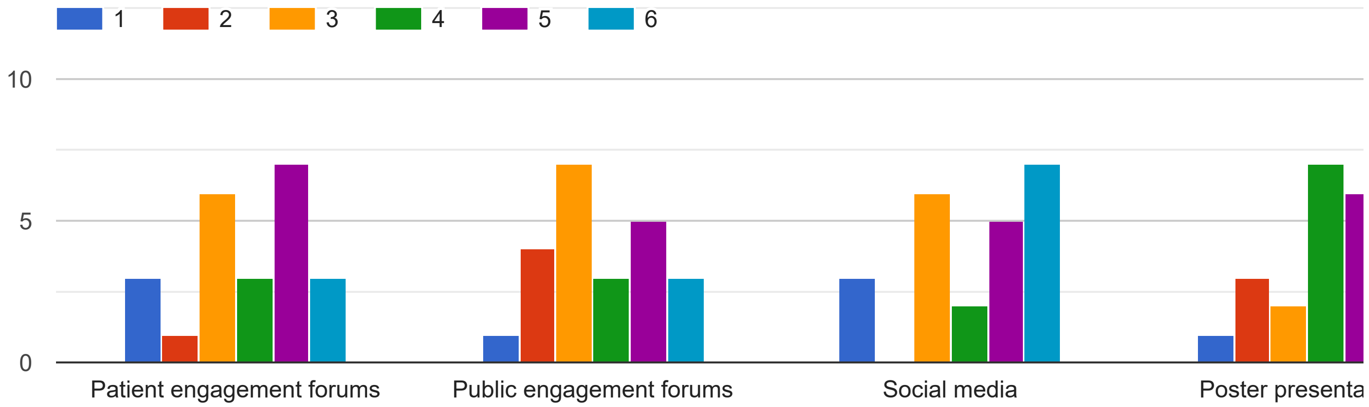

14. Which knowledge translation activities have you participated in the past?

23 responses

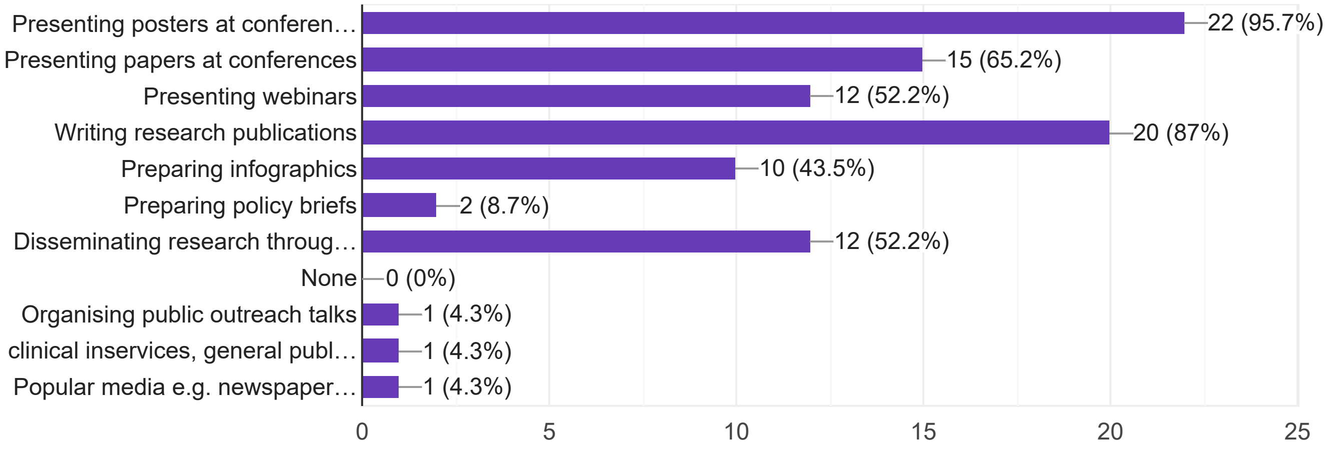

15. Which non-academic research stakeholders have you interacted with in your research and knowledge translation activities?

23 responses

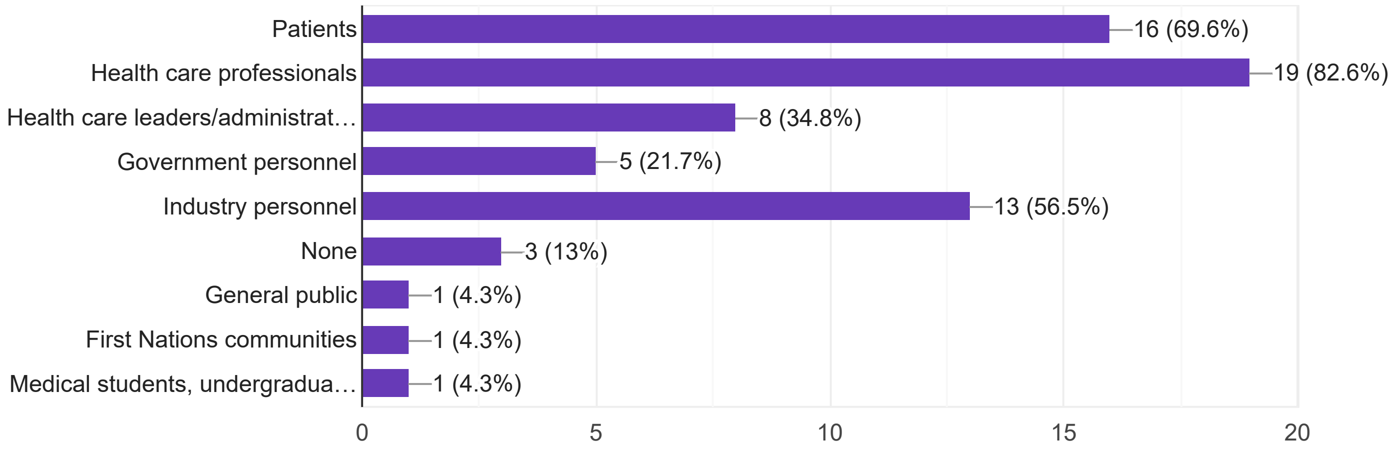

16. Which knowledge translation topics are you familiar with?

23 responses

Mainly standard academic research disemmination

Interactions with healthcare professionals

Presenting posters at conferences

Synthesis of knowledge by involved in a review paper. Also been involves in some KT dissemination work - e.g., poster prestation, paper, webinar, and developing educational pamphlets for patients and caregivers.

Not sure what you mean by this - are you talking about implementation science?

academic, like making poster for conferences

NA

17. Which knowledge translation topics are you less familiar with?

23 responses

Government; policy-makers; patient/health-provider groups

Public engagement

Disseminating research through social media

Exchange of knowledge, evaluation of outcomes, applying knowledge to the real-world, etc.

as above

outside academic

NA

quite a broad question.

18. What topics would you like to see covered in the Knowledge Translation training?

15 responses

Lay language summary writing; focus groups; theoretical framework of KTM

Public/patients engagement

Presentation and writing papers.

All areas, but perhaps with focus on knowledge dissemination to better communicate research to the wider audience.

I'll leave this to the trainees.

Most effective means for different stakeholders

n/a

KT strategies to directly reach the public- similar to direct to consumer marketing

19. What is your learning goal for the Knowledge Translation training program?

23 responses

To become more familiar of, and confident with, KTM strategies

Improve my communication skills in working with non-academic/non-healthcare audience.

Good communication with others.

Gain an overview of the KT process, better communication skills, and identify areas that interests the trainee to advance their careers.

I'll leave this to the trainees.

To know what the knowledge translation is

NA

?

20. Please indicate which knowledge translation resources you are familiar with.

21 responses

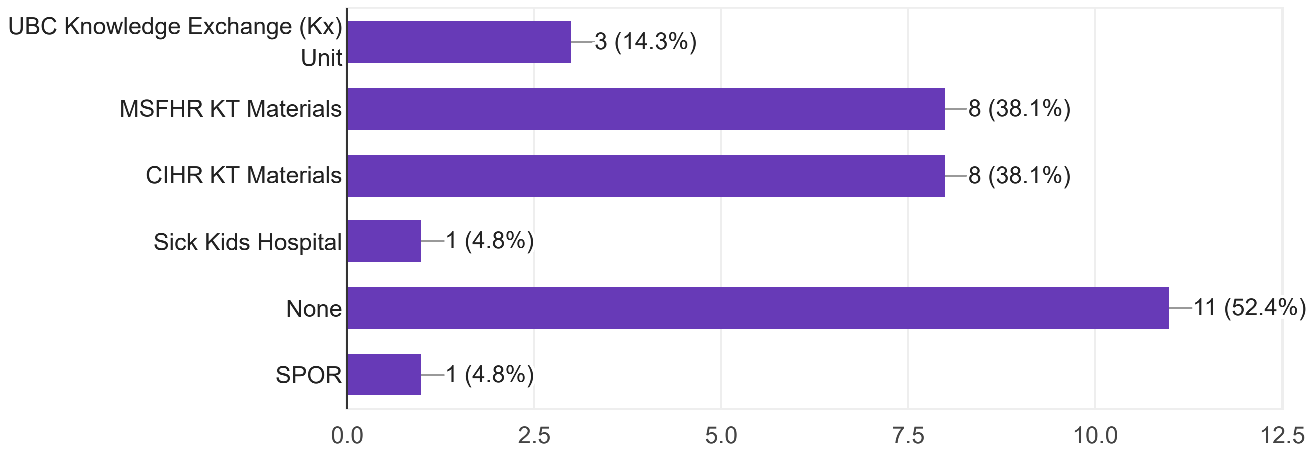

21. Please share any other comments to inform the design of the Knowledge Translation training program.

6 responses

Hands-on, engaged training

good to have an opportunity to learn knowledge translation

Please make the program easier for entry level participants.

I look forward to learning from this program. This could create projects that go beyond academia and truly serve health care professionals to improve the care provided to patients. I would prefer a course format for this program with activities to reinforce what is taught. It can be short quizzes such as those provided by UBC for Biosafety training.

It would be great to make some resource available to trainees for making clear and attractive infographics. Right now I'm using powerpoint which isn't ideal.
