# Supplementary material for: Design, delivery, and evaluation of a knowledge translation intervention for multi-stakeholders
Source: Implement Sci Commun. 2023 Jul 24;4:85. doi: 10.1186/s43058-023-00465-9 (PMC10364428; doi:10.1186/s43058-023-00465-9)
Supplement: Supplementary file 2 — Additional file 2: KT Training Curriculum. [file 43058_2023_465_MOESM2_ESM.docx]

**KTM Training Curriculum**

**Overall Learning Outcomes**

By the end of the Knowledge Translation & Mobilization (KTM) Training, participants will have:

1. Increased knowledge, skills, attitudes, and confidence related to KTM in health research and care.
2. Increased knowledge, skills, attitudes, and confidence in engaging with Indigenous peoples in health research, including history, land acknowledgments, Cultural Safety and Humility, Indigenous ways of knowing, and Indigenous research ethics.
3. Increased knowledge, skills, attitudes, and confidence related to engaging with patients and Patient Family Partners (PFP) in Patient Oriented Research (POR).
4. Improved competencies in building KTM into their research projects through KTM plans.
5. Improved skills in evaluating KTM plans.
6. Improved skills in communicating research to multiple audiences, including non-academic audiences.
7. Increased networking and collaboration competencies with non-academic audiences.
8. Have increased understanding and skills in sharing research knowledge through media.
9. Increased knowledge and skills related to linking with policy and decision-makers.
10. Have increased engagement in their graduate and post-graduate studies.

**Graphic Syllabus**

The Graphic Syllabus below depicts the learning pathway for the KTM training. The curriculum focuses on the *who*, *what*, *when*, *where*, *why*, and *how* of KTM in health research.


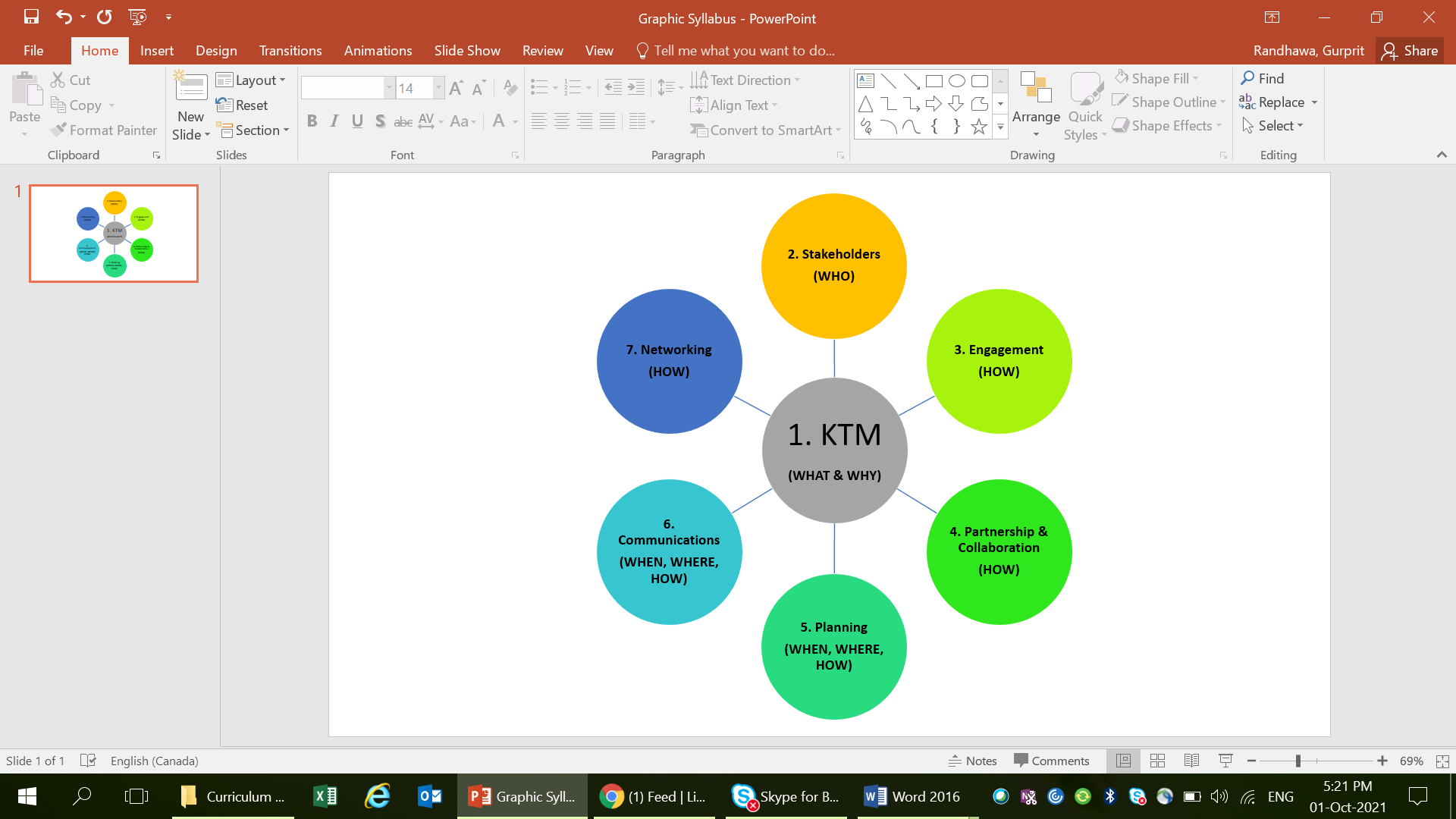


**Detailed Curriculum**

| **Focus Area** | **Topic #** | **Topic** | **Learning Outcomes** | **Delivery Modality** | **Learning Outcome#** |
| --- | --- | --- | --- | --- | --- |
| What | **1** | **KTM in Health Research and Health Care**   - Definition - Why do KTM? | 1. Define KTM 2. Explain the importance of KTM | Webinar  LMS Discussion | 1 |
| Who | **2** | **KTM Stakeholders**   - Who are they? - Stakeholder Needs | 1. Map the assets, decision-making processes, and other factors that shape a system 2. Recognize potential partners, collaborators, and stakeholders with critical expertise 3. Identify and understand stakeholder needs | Presentation | 1 |
| Who | **3** | **Patients**   - Patient Oriented Research - Patient Voices Network - ReachBC | 1. Define POR. 2. Explain the importance of POR. 3. Discuss POR resources. | Presentation and Panel Session | 3 |
| Who | **4** | **Indigenous Peoples**   - History - Land Acknowledgements - Cultural Safety & Humility - Indigenous Research Ethics - Ownership, Control, Access, Possession) - Doing research in a “Good Way” - Indigenous Knowledge Exchange - Two-Spirit Identity | 1. Understand the history of Indigenous peoples, especially as it relates to health care and health research. 2. Increase knowledge, skills, and abilities in providing land acknowledgments. 3. Increase knowledge in indigenous research ethics. 4. Increase understanding of Indigenous ways of knowing and knowledge exchange. 5. Increase understanding of Two-Spirit identity in health research. | Presentation | 2 |
| How | **4** | **KTM Engagement**   - IAP2 Framework - Patient Engagement (Detailed) - Indigenous Engagement - Engaging Knowledge Users | 1. Increase knowledge, skills, and attitudes related to engaging KTM stakeholders, especially patients, Indigenous peoples, and knowledge users. | Presentation | 6 |
| How | **5** | **KTM Partnership & Collaboration** | 1. Develop generative questions that you might ask an existing or potential partner 2. Identify a set of considerations to address when building or maintaining a research partnership | Presentation and Panel Session | 6, 8 |
| When, Where, How | **6** | **Planning**   - KTM Plan - Research Ethics   - Communication   - KTM Plan   - So What | 1. Develop a KTM plan. 2. Prepare a strong research ethics proposal. | Presentation | 4 |
| When, Where, How | **7** | **Communications**   - Plain Language - Media - Social Media - Traditional modalities - Innovation/Creativity | 1. Create a concise piece of content to disseminate. 2. Develop a communication strategy for your intended purpose, audience and context. 3. Prepare an effective conference abstract, conference poster and presentation. | Presentation and Panel Session  3MT Heat Coaching | 5, 7, 8, 9 |
| How | **8** | **KTM Evaluation** | 1. Evaluate a KTM Plan. | Presentation | 5 |
| How | **9** | **KTM Networking**   - Conferences - Public Forums - LinkedIn - Twitter - Career Planning - Coaching - Mentoring | 1. Increase skills in networking with stakeholders. | Panel Session | 7 |
