# Supplementary material for: Design, delivery, and evaluation of a knowledge translation intervention for multi-stakeholders
Source: Implement Sci Commun. 2023 Jul 24;4:85. doi: 10.1186/s43058-023-00465-9 (PMC10364428; doi:10.1186/s43058-023-00465-9)
Supplement: Supplementary file 3 — Additional file 3: KT Training Sessions Summary. [file 43058_2023_465_MOESM3_ESM.docx]

**KT Training Sessions SUmmary**

| **Session (format)** | **Presenter(s)/panelist(s)** | **Title** | **Overview** |
| --- | --- | --- | --- |
| 1. KTM in Health Research and Healthcare (presentation) | Genevieve Creighton - Manager for Knowledge Translation, Michael Smith Health Research BC | Making Research Make a Difference: Using KT Strategies to Move Your Evidence to Impact | Introduction to Knowledge Translation and Mobilization (KTM): definition, purpose, and terminology. KT competencies (activity) using case studies, and description of the KT Pathways digital training and assessment tool for KT knowledge. |
| 2. KTM Stakeholders (presentation) | Dr. Julienne Jagdeo – Health Sector Manager, Genome BC | Stakeholders in Knowledge Mobilization: Who They Are and How They Help Move Evidence into Action | Introduction to stakeholders: who they are, their value and how to identify and prioritise stakeholders. Stakeholder engagement (activity), mapping using an onion diagram, and the power/interest matrix. |
| 3. Patient-Oriented Research (presentation & panel) | Lynne Feehan, Larry Mroz, Rebecca Metcalfe – BC SUPPORT  Karen Rideout – Knowledge Translation Specialist, Legacy for Airway Health  Tony Lanier - Patient-partner | Patient Oriented Research | Introduction to Canada’s Strategy for Patient-Oriented Research (SPOR) to ensure that the right patient receives the right intervention at the right time. Necessity of SPOR – only 15% of funded research gets into practice. Meaningful patient partner engagement and what POR is not. Panel and audience questions for patient-partner (Tony Lanier). |
| 4. Indigenous Peoples (presentation) | Jessica Humchitt – Indigenous Research Analyst, First Nations Health Authority  Katie Bauder – Research Advisor, First Nations Health Authority | Furthering Our Shared Understandings of Indigenous Knowledge Exchange | Indigenous perspectives and knowledge exchange factors. The importance of positionality and its relationship to cultural safety and humility. Considerations for community-driven knowledge exchange in clinical settings. |
| 5. Stakeholder Engagement (presentation) | Maria Hudspith – Executive Director, Pain BC  Linda Wilhelm – Patient-partner | Engagement of People with Lived Experience | Definition of and impetus for patient engagement in individual care, program and service design or research, and system and governance. What patient engagement is not. Patient engagement: benefits, CIHR guidelines, principles of engagement, spectrum, critical steps, roles, case studies and roles. |
| 6. KTM Partnership and Collaboration (presentation & panel) | Naomi Potter (host) - Research Assistant, Center for Heart Lung Innovation KTM Project  Kristi Coldwell - Patient-partner | Effective Partnership and Collaboration | What partnership and collaboration is and why it is important. How to effectively collaborate and maintain relationships. The art of powerful questions and applying empathy to them – empathy map. Panel and audience questions for patient partner (Kristi Coldwell). |
| 7. KTM Planning (presentation) | Stephanie Warner – Manager of Knowledge Engagement, University of Calgary  Alyse Pearce – Specialist in Knowledge Engagement, University of Calgary | Knowledge Mobilization Planning and Strategy | Knowledge engagement, mobilization, and planning. Understanding and matching activities to stakeholders and target audiences – Wheel of Involvement. Selecting appropriate levels of engagement. Knowledge mobilization activities: types, timing, and evaluation. Writing a knowledge mobilization plan and integrating it into a funding application. Getting to impact. |
| 8. Communication in KTM (panel) | Barb Langlois – Program Director of Surgery, Providence Health Care  Christine Lyon – Communications Specialist, Providence Health Care  Maryke Peter – Paralegal and student at the Center for Heart Lung Innovation | Best Practices in KTM Communication | The science news cycle, types of news stories, and media techniques. Proactive and reactive news pitches (examples). What makes a story newsworthy? Plain language communication in policy, governance, and litigation (with case studies). Strategy for engagement. |
| 9. KTM Evaluation (presentation) | Aggie Black - Director of Research and Knowledge Translation, Providence Health Care | How can you tell if your KT plan is working? Things we’ve learned from the Knowledge Translation Challenge. | The KT Challenge program – description, evaluation of findings, and ideas on how to evaluate KT plans. Typical activity indicators to track (SMART indicators) and data collection tools. |
| 10. Networking in KTM (panel) | Dr. Gurprit Randhawa (host) - Project Coordinator, Center for Heart Lung Innovation KTM Project  Genevieve Creighton - Manager for Knowledge Translation, Michael Smith Health Research BC  Stephanie Warner – Manager of Knowledge Engagement, University of Calgary  Aggie Black - Director of Research and Knowledge Translation, Providence Health Care | KTM Networking Panel | Q & A about networking and careers in KTM. |
| 11. KTM Planning Workshop (workshop) | Dr. Gurprit Randhawa - Project Coordinator, Center for Heart Lung Innovation KTM Project | Building Your KTM Plan | Detailed run through and hands-on application of the Knowledge Translation Planning Template © from The Hospital for Sick Children |
