# Supplementary material for: Design, delivery, and evaluation of a knowledge translation intervention for multi-stakeholders
Source: Implement Sci Commun. 2023 Jul 24;4:85. doi: 10.1186/s43058-023-00465-9 (PMC10364428; doi:10.1186/s43058-023-00465-9)
Supplement: Supplementary file 4 — Additional file 4: Data Collection Tools. [file 43058_2023_465_MOESM4_ESM.docx]

**Data Collection Tools**

**Knowledge Translation and Mobilization: Reimagining Graduate Student Education to Create the Next Generation of Health Professionals, Advocates and Communicators**

INITIAL ASSESSMENT SURVEY (TRAINEES)

This survey is aimed at assessing your knowledge translation and mobilization (KTM) experience and competence before participating in the KTM training sessions. It should take approximately 5-15 minutes to complete.

Demographic information

Q1. Please fill in your email address: __________________

Q2. Please indicate your role:

- Master’s Student
- PhD Student
- MD/ PhD Student
- Post-Doctoral Fellow
- Other ____________

KTM competence initial assessment

Q3. Please rate your current confidence in carrying out knowledge translation and mobilization (KTM) activities.

Extremely unconfident - 1 2 3 4 5 6 7 8 9 10 - Extremely confident

Q4. Please rate your current knowledge on the topic of KTM.

Extremely unknowledgeable - 1 2 3 4 5 6 7 8 9 10 - Extremely knowledgeable

Q5. Please rate your current attitude toward KTM.

Extremely negative- 1 2 3 4 5 6 7 8 9 10 - Extremely positive

Q6. Please rate your current ability to build KTM into your research projects.

Extremely unable - 1 2 3 4 5 6 7 8 9 10 - Extremely able

Q7. Please rate your skills in creating a KTM plan.

Extremely unskilled - 1 2 3 4 5 6 7 8 9 10 - Extremely skilled

Q8. Please rate your skills in evaluating a KTM plan.

Extremely unskilled - 1 2 3 4 5 6 7 8 9 10 - Extremely skilled

Q9. Please rate your current competence in communicating with non-academic research stakeholders.

Extremely low - 1 2 3 4 5 6 7 8 9 10 - Extremely high

Q10. Please rate your current competence in collaborating with non-academic research stakeholders.

Extremely low - 1 2 3 4 5 6 7 8 9 10 - Extremely high

Q11. Please rate your current competence in networking with non-academic research stakeholders.

Extremely low - 1 2 3 4 5 6 7 8 9 10 - Extremely high

Q12. Please rate your level of engagement in your graduate and post-graduate studies.

Extremely unengaged - 1 2 3 4 5 6 7 8 9 10 - Extremely engaged

Q13. Please rate your perceived level of competitiveness for KTM funding opportunities.

Extremely uncompetitive - 1 2 3 4 5 6 7 8 9 10 - Extremely competitive

Q14. Please rate your perceived level of competitiveness for the current job market.

Extremely uncompetitive - 1 2 3 4 5 6 7 8 9 10 - Extremely competitive

Q15. Please rate your likelihood of applying to UBC Public Scholars Initiative (PSI) and other “Re-imagining the PhD” opportunities?

Extremely unlikely - 1 2 3 4 5 6 7 8 9 10 - Extremely likely

Q16. Is there anything else that you would like to share?

[open-ended]

PROGRAM FOLLOW-UP ASSESSMENT SURVEY – TRAINEES

This survey is aimed at assessing your knowledge translation and mobilization (KTM) experience and competence after participating in the KTM training sessions. It should take approximately 5-15 minutes to complete.

Demographic information

Q1. Please fill in your email address: __________________

Q2. Please indicate your role:

- Master’s Student
- PhD Student
- MD/ PhD Student
- Post-Doctoral Fellow
- Other ____________

Q3. Please indicate the KTM sessions that you attended.

- KTM in Health Research and HealthCare
- KTM Stakeholders
- Patient/ Family Partners
- Indigenous Peoples
- KTM Engagement
- Partnership & Collaboration
- Planning
- Communications
- KTM Evaluation
- KTM networking
- None

KTM experience/ knowledge follow-up assessment

Q4. Please rate your current confidence in carrying out knowledge translation activities.

Extremely unconfident - 1 2 3 4 5 6 7 8 9 10 - Extremely confident

Q5. Please rate your current knowledge on the topic of KTM.

Extremely unknowledgeable - 1 2 3 4 5 6 7 8 9 10 - Extremely knowledgeable

Q6. Please rate your current attitude toward KTM.

Extremely negative - 1 2 3 4 5 6 7 8 9 10 - Extremely positive

Q7. Please rate your current ability to build KT into your research projects.

Extremely unable - 1 2 3 4 5 6 7 8 9 10 - Extremely able

Q8. Please rate your skills in creating a KTM plan.

Extremely unskilled - 1 2 3 4 5 6 7 8 9 10 - Extremely skilled

Q9. Please rate your skills in evaluating a KTM plan.

Extremely unskilled - 1 2 3 4 5 6 7 8 9 10 - Extremely skilled

Q10. Please rate your current competence in communicating with non-academic research stakeholders.

Extremely low - 1 2 3 4 5 6 7 8 9 10 - Extremely high

Q11. Please rate your current competence in collaborating with non-academic research stakeholders.

Extremely low - 1 2 3 4 5 6 7 8 9 10 - Extremely high

Q12. Please rate your current competence in networking with non-academic research stakeholders.

Extremely low - 1 2 3 4 5 6 7 8 9 10 - Extremely high

Q13. Please rate your level of engagement in your graduate and post-graduate studies.

Extremely unengaged - 1 2 3 4 5 6 7 8 9 10 - Extremely engaged

Q14. Please rate your level of competitiveness for KTM funding opportunities.

Extremely uncompetitive - 1 2 3 4 5 6 7 8 9 10 - Extremely competitive

Q15. Please rate your level of competitiveness for the current job market.

Extremely uncompetitive - 1 2 3 4 5 6 7 8 9 10 - Extremely competitive

Q16. Please rate your likelihood of applying to UBC Public Scholars Initiative (PSI) and other “Re-imagining the PhD” opportunities?

Extremely unlikely - 1 2 3 4 5 6 7 8 9 10 - Extremely likely

Q17. What are your main learning takeaways from this training program?

[open-ended]

Q18. How will you apply your learning takeaways in your work?

[open-ended]

Q19. Is there anything else you would like to share?

[open-ended]

**Stay engaged**

**We would like to know more about your experiences!**

We are recruiting volunteers for a focus group about the KTM program and would like to hear about your experiences. Please contact [Gurprit.Randhawa@hli.ubc.ca](mailto:Gurprit.Randhawa@hli.ubc.ca) for more information.

SESSION FOLLOW-UP ASSESSMENT SURVEY - TRAINEES

This survey is aimed at assessing your satisfaction and learning from this knowledge translation and mobilization (KTM) training session. It should take approximately 5-15 minutes to complete.

Demographic information

Q1. Please fill in your email address: __________________

Q2. Please indicate your role:

- Master’s Student
- PhD Student
- MD/ PhD Student
- Post-Doctoral Fellow
- Other ____________

KTM training session satisfaction assessment

Q3. How satisfied are you with today’s session?

1 = Very Dissatisfied, 2 = Somewhat Dissatisfied, 3 = Neutral, 4 = Somewhat Satisfied, 5 = Very Satisfied

Q4. How satisfied are you with the enrollment process for the session?

1 = Very Dissatisfied, 2 = Somewhat Dissatisfied, 3 = Neutral, 4 = Somewhat Satisfied, 5 = Very Satisfied

Q5. How satisfied are you with the platform of the session?

1 = Very Dissatisfied, 2 = Somewhat Dissatisfied, 3 = Neutral, 4 = Somewhat Satisfied, 5 = Very Satisfied

Q6. How satisfied are you with the content of the session?

1 = Very Dissatisfied, 2 = Somewhat Dissatisfied, 3 = Neutral, 4 = Somewhat Satisfied, 5 = Very Satisfied

Q7. How satisfied are you with the level of engagement of the session?

1 = Very Dissatisfied, 2 = Somewhat Dissatisfied, 3 = Neutral, 4 = Somewhat Satisfied, 5 = Very Satisfied

Q8. How satisfied are you with the speaker/ facilitator’s delivery?

1 = Very Dissatisfied, 2 = Somewhat Dissatisfied, 3 = Neutral, 4 = Somewhat Satisfied, 5 = Very Satisfied

Q9. How satisfied are you with the organization/ structure of the session?

1 = Very Dissatisfied, 2 = Somewhat Dissatisfied, 3 = Neutral, 4 = Somewhat Satisfied, 5 = Very Satisfied

KTM competence session follow-up assessment

Please rate to which extent you agree or disagree with the following statements (1=Strongly Disagree, 2 = Somewhat Disagree, 3 = Neutral, Somewhat Agree, 5=Strongly Agree)

Q10. This session increased my knowledge of the subject matter.

1 = Strongly Disagree, 2 = Somewhat Disagree, 3 = Neutral, Somewhat Agree, 5=Strongly Agree

Q11. This session increased my skills in the subject matter.

1 = Strongly Disagree, 2 = Somewhat Disagree, 3 = Neutral, Somewhat Agree, 5=Strongly Agree

Q12. This session enhanced my attitude towards the subject matter.

1 = Strongly Disagree, 2 = Somewhat Disagree, 3 = Neutral, Somewhat Agree, 5=Strongly Agree

Q13. This session enhanced my confidence in the subject matter.

1 = Strongly Disagree, 2 = Somewhat Disagree, 3 = Neutral, Somewhat Agree, 5=Strongly Agree

Q14. This session provided content that is relevant to what I am expected to do in my current role.

1 = Strongly Disagree, 2 = Somewhat Disagree, 3 = Neutral, Somewhat Agree, 5=Strongly Agree

Q15. The session was relevant to what I might be expected to do in my future role/job.

1 = Strongly Disagree, 2 = Somewhat Disagree, 3 = Neutral, Somewhat Agree, 5=Strongly Agree

Q16. I would recommend this session to others.

1 = Strongly Disagree, 2 = Somewhat Disagree, 3 = Neutral, Somewhat Agree, 5=Strongly Agree

Additional questions

Q17. What did you like about the session?

[open-ended]

Q18. Do you have any suggestions for improvements?

[open-ended]

Q19. What is your main takeaway from this session and how will you apply it in your work?

[open-ended]

Q20. We are organizing more KTM sessions and resources. Which of these sessions and resources are you interested in?

□ KTM in Health Research and HealthCare

□ KTM Stakeholders

□ Patient/ Family Partners

□ Indigenous Peoples

□ KTM Engagement

□ Partnership & Collaboration

□ Planning

□ Communications

□ KTM Evaluation

□ KTM networking

Q21. Is there anything else you would like to share?

[open-ended]

PROGRAM FOLLOW-UP ASSESSMENT SURVEY - PARTNERS

This survey is aimed at getting your perspectives on the knowledge translation and mobilization (KTM) training program. It should take approximately 1 - 5 minutes to complete.

Q1. Please indicate the KTM sessions in which you partnered with us.

- KTM in Health Research and HealthCare
- KTM Stakeholders
- Patient/ Family Partners
- Indigenous Peoples
- KTM Engagement
- Partnership & Collaboration
- Planning
- Communications
- KTM Evaluation
- KTM networking
- Other: _________________

KTM partner satisfaction follow-up assessment

Q2. How satisfied are you with the KTM training session(s) that you partnered on?

1 = Very Dissatisfied, 2 = Somewhat Dissatisfied, 3 = Neutral, 4 = Somewhat Satisfied, 5 = Very Satisfied

Q3. How satisfied are you with the information you received in preparation for the KTM training session(s)?

1 = Very Dissatisfied, 2 = Somewhat Dissatisfied, 3 = Neutral, 4 = Somewhat Satisfied, 5 = Very Satisfied

Q4. How satisfied are you with the support you received in preparation for the KTM training session(s)?

1 = Very Dissatisfied, 2 = Somewhat Dissatisfied, 3 = Neutral, 4 = Somewhat Satisfied, 5 = Very Satisfied

Q5. How likely are you to recommend the KTM training session(s) to someone else?

1 = Very Unlikely, 2 = Somewhat Unlikely, 3 = Neutral, 4 = Somewhat Likely, 5 = Very Likely

Q6. How likely are you to collaborate with us in the future?

1 = Very Unlikely, 2 = Somewhat Unlikely, 3 = Neutral, 4 = Somewhat Likely, 5 = Very Likely

Q7. How likely are you to recommend this partnership to someone else?

1 = Very Unlikely, 2 = Somewhat Unlikely, 3 = Neutral, 4 = Somewhat Likely, 5 = Very Likely

Q8. What did you like most about this partnership?

[open-ended]

Q9. What were the strengths of our partnership?

[open-ended]

Q10. Do you have any suggestions for improvements in future?

[open-ended]

Q11. Is there anything else that you would like to share?

[open-ended]

PROGRAM FOLLOW-UP FOCUS GROUP - TRAINEES

This focus group is aimed at getting your perspectives on the knowledge translation and mobilization (KTM) training program. It should take approximately 45 - 60 minutes to complete.

Q1. What comes to mind when you hear “knowledge translation & mobilization”? Probe: How would you define knowledge translation and mobilization?

Q2. Please share an aspect of your work that interested you in the KTM training program?

Q3. How satisfied were you with the KTM training program?

Q4. What did you like most about the KTM training program?

Q5. How can the KTM training program be improved?

Q6. Which stakeholders have you previously worked with?

Q7. Did the KTM training program improve your competence in working (communicating, collaborating, networking) with non-academic stakeholders? Please explain.

Q8. How has the KTM training program impacted your level of engagement with your graduate and post-graduate studies? Please explain.

Q9. What was the most important concept or skill you learned from this KTM program and how do you intend to apply it in your own work?

Q10. What surprised you about the KTM program?

Q11. Would you recommend this KTM training program to other people? Please explain.

Q12. Is there anything else that you would like to share?

PROGRAM FOLLOW-UP FOCUS GROUP – FACULTY

This focus group is aimed at getting your perspectives on the knowledge translation and mobilization (KTM) training program. It should take approximately 45 - 60 minutes to complete.

Q1. What comes to mind when you hear “knowledge translation & mobilization”? Probe: How would you define knowledge translation and mobilization?

Q2. Please share an aspect of your work that interested you in the KTM training program?

Q3. Were you satisfied with the KTM training program?

Q4. What did you like most about the KTM training program?

Q5. How can the KTM training program be improved?

Q6. How did the KTM training program affect your engagement with stakeholders to enhance knowledge mobilization in your research?

Q7. How did the KTM program contribute to providing a well-rounded training environment for your trainees? Please explain.

Q8. How satisfied are you with your trainees’ scholarly performance?

Q9. Do you think the KTM training program could improve your trainees’ scholarly performance? How so?

Q10. What surprised you about the KTM program?

Q11. What surprised you about your trainees who attended the KTM program?

Q12. Would you recommend this KTM training program to other people? Please explain.

Q13. Is there anything else that you would like to share?

PROGRAM FOLLOW-UP FOCUS GROUP – PUBLIC AND PATIENT/FAMILY PARTNERS

This focus group is aimed at getting your perspectives on the knowledge translation and mobilization (KTM) training program. It should take approximately 45 - 60 minutes to complete.

Q1. What comes to mind when you hear “knowledge translation & mobilization”? Probe: How would you define knowledge translation and mobilization?

Q2. Please share an aspect of your work or life that interested you in the KTM training program?

Q3. Were you satisfied with the Patient and Family forum(s)?

Q4. What did you like most about the Patient and Family Forum?

Q5. How has participating in the Patient and Family Forum impacted your sense of empowerment as a patient?

Q6. How can the Patient and Family Partner Forums be improved?

Q7. How can KTM be improved?

Q8. Would you recommend this Patient and Family Forums to other people? Please explain.

Q9. Is there anything else that you would like to share?
