# Supplementary figures and images for: Design, delivery, and evaluation of a knowledge translation intervention for multi-stakeholders
Source: Implement Sci Commun. 2023 Jul 24;4:85. doi: 10.1186/s43058-023-00465-9 (PMC10364428; doi:10.1186/s43058-023-00465-9)

**Data Collection Plan**


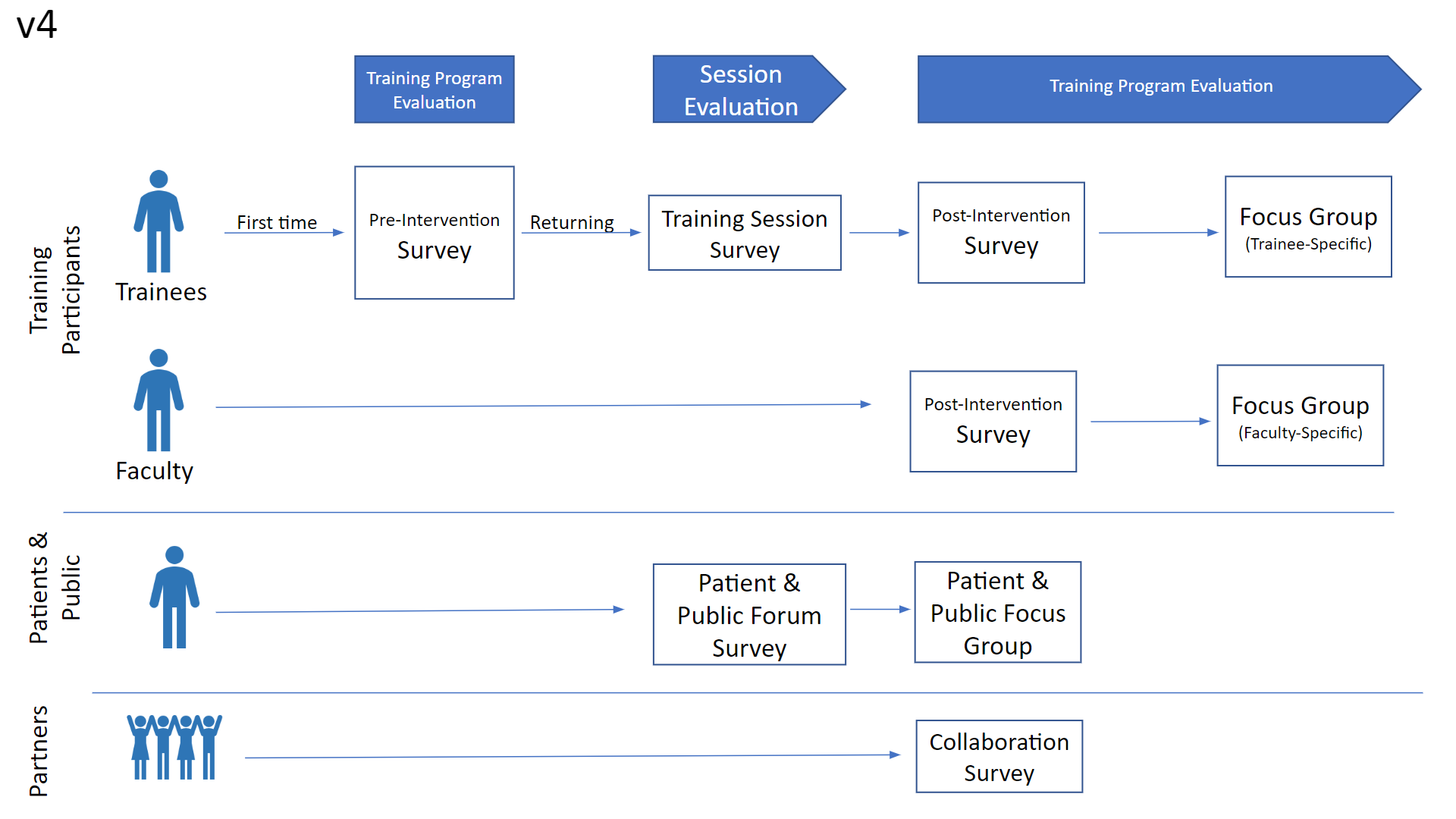

Supplement: Supplementary file 5 — Additional file 5: Data Collection Plan. [file 43058_2023_465_MOESM5_ESM.docx]
